# Comprehension of the age-dependent gut and brain interaction of honey bee workers by integration of multi omics approaches

**DOI:** 10.1016/j.jare.2025.07.045

**Published:** 2025-07-30

**Authors:** Cassandra Uthoff, Yelyzaveta Yakovlyeva, Beatrice Engelmann, Ulrike Rolle-Kampczyk, Sven-Bastiaan Haange, Abdulrahim T. Alkassab, Jens Pistorius, Fynn Brix, Silvio Waschina, Andreas S. Thum, Nico Jehmlich, Martin von Bergen

**Affiliations:** a Department of Molecular Toxicology, Helmholtz Centre for Environmental Research – UFZ GmbH, Leipzig 04318, Germany; b Federal Research Centre for Cultivated Plants, Institute for Bee Protection, Julius Kühn- Institut (JKI), Braunschweig 38104, Germany; c Christian-Albrechts-University, Kiel, Germany; d Abteilung für Lebensmitteltechnologie, Institut für Humanernährung und Lebensmittelkunde, Kiel 24105, Germany; e Nutriinformatics Research Group, Institute for Human Nutrition and Food Science, Kiel University, Kiel 24105, Germany; f Institute of Biology, Leipzig University, Leipzig 04103, Germany; g Institute of Biochemistry, Leipzig University, Leipzig 04103, Germany; h German Centre for Integrative Biodiversity Research (iDiv) Halle-Jena-Leipzig, Puschstraße 4, Leipzig 04103, Germany

**Keywords:** Host-microbiome interactions, *Apis mellifera*, Microbiome, Metabolomics, Meta-proteomics, Division of labour

## Abstract

**Introduction:**

In honey bees, division of labour is a key feature, with age-related behavioural transitions being closely associated with molecular changes in the brain, gut, and microbiota. Despite evidence of both microbiome and brain changes in honey bees, most studies focus on either aspect or a single method of investigation, limiting our understanding of their interconnected roles in development and task differentiation.

**Objectives:**

In this study, we investigated the molecular changes in the gut and brain in honey bee workers of different ages using (meta-)proteomics and metabolomics to better understand their contribution to behavioural responses and modulation.

**Methods:**

Workers were sampled at seven timepoints throughout their life. Mass spectrometry for (meta-) proteomic and metabolomic of the guts and brains allowed insights into the global structural and functional dynamics of the microbiota, as well as the functional and metabolic alterations in the host gut and brain, and their interactions.

**Results:**

Our results indicate the transport of amino acids between the gut and brain, potentially influencing functional pathways and behavioural phenotypes. We observed a correlation between concentrations of tryptophan and its metabolic products between honey bee brain and gut. This provides evidence regarding gut-brain axis as a way of internal communication for different host mechanisms in honey bees. Microbiota composition changed significantly, with protein numbers increasing significantly in the establishment phase. Proteomic results from both the host and the microbiota reveal that altered metabolic and functional pathway abundances may be due to energy expenditure, task differentiation, and age of onset of foraging.

**Conclusion:**

Overall, our findings are the first to describe the global (meta-)proteomic and metabolomic changes in the honeybee gut and brain throughout a worker’s life. This provides new insights toward developing potential biomarkers for evaluation of different functional changes related to various environmental stressors.

## Highlights

• Majority of microbiome, metabolomic, and proteomic changes occurred during the establishment phase.
• Pathway alterations suggest links to energy expenditure and task differentiation.
• Tryptophan and its metabolic products exhibit correlation between the gut and brain of workers.
• Possible amino acids transport between gut and brain may influenced by key functional pathways.

## Graphical abstract

**Figure:**
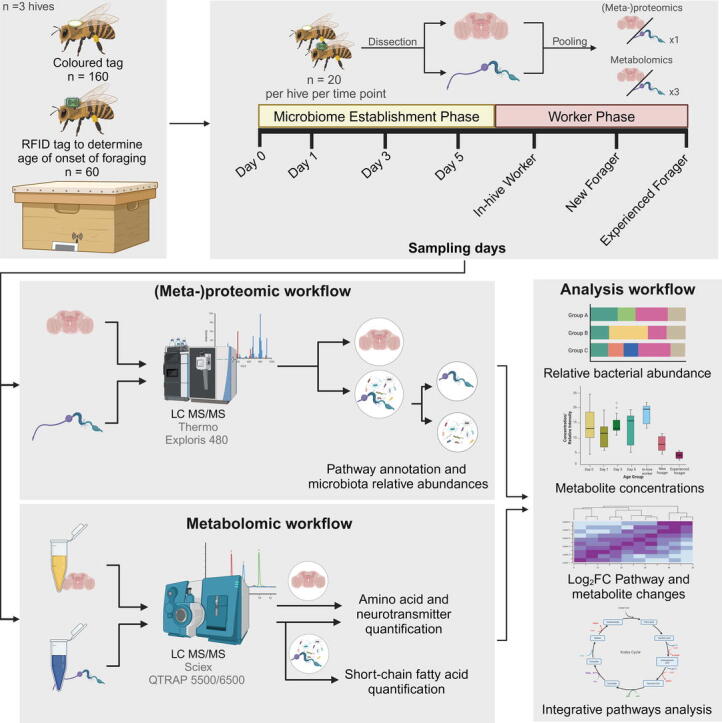

## Introduction

Social insects often exhibit division of labour based on worker size [1,2]. In many ant species, workers are morphologically adapted for specific roles such as brood tending, foraging or colony defence [3]. In contrast, bumblebees divide tasks solely based on worker size, with smaller bees focusing on in-hive tasks and larger ones responsible for foraging [4]. Honey bee workers (*Apis mellifera*), on the other hand, divide tasks according to age. In the first days of their lives, workers are responsible for cleaning, followed by nursing behaviours in the days after. Older bees take on a plethora of different roles such as queen attendance, nectar reception and guarding, depending on colony requirements [5]. After about two to three weeks, in-hive behaviour transitions into foraging [5]. This worker development is accompanied by internal molecular changes, such as changes in insect juvenile hormone levels and enzyme activity, both of which have been directly linked to age and task differentiation [[6], [7], [8]].

The idea that the microbiome and host are intricately linked via the gut-brain axis has recently gained a lot of interest [[9], [10], [11]]. It has been suggested that the microbiota plays an important role in nutrition regulation, immune function, and behaviour via cross-communication [12]. Additionally, the microbiome affects insect learning and memory, and has been used to enhance comprehension of the phenotype and development of neurodegenerative diseases [9].Honey bees are an effective model for studying host-microbiome interactions, as they harbour a simple yet stable bacterial community of 8–10 phylotypes [13,14]. Age-matched workers can be used to control for different developmental and behavioural stages, while dissection of gut and brain tissues allows for targeted analyses. Workers can be reared to be microbiome-free by preventing contact with older workers or the hive surface. Controlled mono- or multi-colonisation with different bacteria can elucidate the specific functions of each phylotype and its interactions with other species or the host [9]. For instance, *Gilliamella apicola* and *Snodgrassella alvi* form a biofilm in the ileum and engage in cross-feeding. *G. apicola* accumulates pyruvate, which *S. alvi* then metabolises, and the produced amino acids can be utilised by *Bombilactobacillus* spp. (previously known as Firm-4) [15]. The presence of *Bombilactobacillus* spp.*, Lactobacillus* spp. (previously known as Firm-5) and *Bifidobacterium* spp. is essential for the expression of genes involved in caste determination and age polyethism (reviewed in Motta and Moran [14]), highlighting the microbiome’s critical role in host development.

The honey bee brain undergoes structural modifications during behavioural transitions, with foragers exhibiting an expansion in specific brain areas such as mushroom body subregions, attributed to the reorganization of cytoskeletal components [16,17]. Gene expression shifts during maturation are also linked to foraging behaviour, reflecting internal adaptations that align with the worker’s evolving tasks [18,19]. Although evidence suggests changes in both microbiome and the brain, most studies focus on one of these aspects, often using a single analytical approach. While this provides detailed insights into specific functions within particular age groups, it often lacks a connection to the microbiome and its interactions in the gut and brain.

The current study aims to integrate metabolomic and (meta-) proteomic approaches, facilitating the understanding of how structure, function, and metabolic activity interact in honey bee workers throughout their different life stages. By sampling workers at seven different time points, we seek to investigate the (1) changes in the structure and function of the microbiota, and its interaction with the host; (2) the variations in amino acid and biogenic amine concentration in the brain, and their potential involvement in the division of labour; and (3) the correlation between metabolite levels in the gut and brain to determine candidates that may be transported along the gut-brain axis. All objectives refer to changes with increasing age of workers, and specifically the difference between workers with a developing and fully developed microbiome (establishment and worker phase respectively). This will provide a comprehensive view of host-microbiome interactions on a molecular level, paving the way for further targeted studies on these interactions.

## Materials and methods

### Rearing and sampling of honey bee workers

Honey bee workers for this study were collected from honey bee colonies (Buckfast strain) located at the Helmholtz Centre for Environmental Research, Leipzig (51°21′14.3″N, 12°25′57.6″E). The bees for the experiment were kept in three Dadant US observation hives (48.2x44.8x28.5 cm, Bienen Janisch, Hartl, Austria) to simplify the sampling process of the marked honey bees as well as the search for tagged and numbered forager bees. Colonies were established one week before the start of the experiment. Two frames, one of which had the queen on it, were taken out of a full sized Dadant US colony and placed into the observation hive. Both frames were placed on top of one another, with the bottom one composed of approximately 75 % brood, whereas the top frame acted as food storage. Thus, each colony comprised 4000–5000 workers and a fertile 1-year-old sister queen to decrease genetic variability. Bees were placed on the edge of the Environmental Research Centre campus, where they had access to flowering trees, as well as a range of small allotments close to the study site, ensuring sufficient nectar and pollen influx. After the initial establishment, the colonies were examined by the beekeeper only though the glass of the observation hive, to decrease stress and allow for proper acclimatisation. The setup and sampling were done by a qualified beekeeper in accordance to good beekeeping practice.

To obtain age-matched bees, newly emerged workers (NEWs) were reared by collecting three Dadant frames from the original hives used to establish the observation hives, with a large area of capped brood. Those were placed in an incubator at 35 °C and 80 % humidity in the afternoon and left overnight. On the next day, NEWs were collected from the frames and marked on the thorax with a coloured dot (POSCA pens, Heinrich Holtermann KG, Brockel, Germany) with each colour representing one hive. One hundred and sixty workers were marked for each hive.

Additionally, to record the age of onset of foraging to differentiate between new and experienced foragers, 60 more bees per hive were tagged with RFID tags (Microsensys GmbH, Erfurt, Germany). The bees were additionally marked with an individual number using the paint-dot system [20], a method of numbering bees using colour and location of the paint dots on the worker’s thorax. This allowed us to monitor and sample individual workers, depending on their foraging activity. To differentiate workers with the same number between different hives, the bees were also marked with a small coloured dot at the end of their abdomen according to their hive colour.

After taking Day 0 samples from the frames in the incubator (n = 20 for each hive), the remaining marked workers were directly introduced to their respective observation hives. Bees were placed in the empty feeding compartment, with a thin tissue placed over the hole leading into the hive. This allowed for slow entrance of the new workers to their hives and decreased the rate of rejection by workers inside the hive. Following the successful integration of the NEWs on Day 0, 20 colour-marked bees from each hive were collected on Days 1, 3 and 5 to cover the establishment phase of the gut microbiota [21]. In-hive workers were defined as workers inside the hives that have a fully developed microbiome. To cover multiple days of the in-hive worker stage, we collected five bees per hive every day from Day 7 to Day 10, equating to a total of 20 workers per hive.

For the foraging phase, we differentiated between new and experienced foragers, as these show different behaviour due to different levels of experience, which are linked to learning and memory [22]. As workers differ in their age of onset of foraging, an RFID reader (Microsensys GmbH, Erfurt, Germany) was installed in front of each flight hole, forcing the bees to walk through it as they left and entered the hive. Every day, data from these readers were inspected, and the age of onset of foraging was recorded for each tagged bee. Based on experience, they were either collected one to two days after onset (new foragers) or at least four days after onset (experienced foragers). As for all other stages, 20 workers from each stage were taken per hive. An exception to this is Hive 1, where the RFID reader was faulty, and thus we only collected four workers for each foraging stage.

All sampled bees were placed directly in liquid nitrogen to prevent changes in the metabolic profile. They were then decapitated in the laboratory so that the head and body of each bee were kept in a separate Eppendorf tube (Eppendorf, Hamburg, Germany) for later dissection. All samples were stored at −80 °C.

### Preparation and dissection of worker bee samples

From the 20 bees at each time point per hive, workers were pooled to obtain a final sample number of n = 5. Five workers were dedicated to proteomic analyses, with single brains and guts measured and analysed. For the remaining 15 samples, three samples were always pooled for metabolomic analyses. The brain(s) and gut(s) from the same workers were assigned the same sample names, so that correlations between brain and gut happened on an individual/pool level. In total, we had 15 samples from each age group, with the exception of the foraging stages, where owing to the lack of foragers from Hive 1, we only had 11.

Worker guts were dissected on Petri dishes (LABSOLUTE®, TH Geyer GmbH & Co. KG, Renningen, Germany). Sternite 6 and the stinger apparatus were gently pulled using tweezers (Dumont® size 3 and 5, TH Geyer GmbH & Co. KG, Renningen, Germany), which allowed extraction of the entire gastrointestinal tract. The stinger apparatus and venom sac were carefully excised and the honey crop was removed.

Honey bee brains were dissected using a dissecting microscope (SZX7, Olympus, EVIDENT, Tokyo, Japan). The head was placed on a petri dish lined with SYLGARD^TM^ 184 silicone (Sigma-Aldrich, Merck, Darmstadt, Germany). Before pinning the head for dissection, antennae and mouthpieces were removed. Subsequently, double-distilled water (ddH_2_O) was added until the head was fully submerged to facilitate dissection. The head cuticle was removed, and the hypopharyngeal glands and trachea were extracted from the inside. The brain was then separated from the cuticle and remaining tracheal remnants were removed before transferring the clean brain to a new Eppendorf tube.

### Metabolomic measurements

All gut pools were weighed individually while the average weight of the brain samples (10.2 mg per pool) was calculated. Each pool was mixed with five times the volume (in µL) of acetonitrile (ACN): H_2_O (1:1, (v/v)) and homogenized using a TissueLyser II (30 Hz, 10 min; Retsch Qiagen, Hilden, Germany). After centrifugation (2 min, 14,000 rpm; Centrifuge 5430 R, Eppendorf, Hamburg, Germany), 10 µL and 40 µL were used for amino acid and SCFA derivatization, respectively.

For SCFA derivatization, the method of Han et al. [23] was modified. The supernatant was combined with 20 µL of 3-nitrophenylhydrazine in 50 % ACN (200 mM) and 20 µL of N-(3-dimethylaminopropyl)-N‘-ethylcarbodiimide hydrochloride in 50 % ACN with 6 % pyridine (120 mM) and incubated (30 min at 40 °C) in a thermomixer (Eppendorf, Hamburg, Germany).

Prior to measurement, the derivative was diluted 1:50 in 10 % ACN. Each sample (10 µL) was injected onto the RSLC UltiMate 3000® system (ThermoFisher Scientific™, Waltham, MA, USA) coupled online to a QTRAP® 5000 mass spectrometer (Sciex, Framingham, USA). SCFA were separated on an Acquity UPLC BEH C18 column (1.7 µm, 2.1 x 100 mm) with A: H_2_O + 0.01 % formic acid and B: ACN + 0.01 % formic acid as mobile phases. The constant flow rate was set to 0.35 mL and the column oven was set to 40 °C. The chromatographic gradient was as follows: 0–2 min at 15 % B, 2–15 min 15–50 % B, 15–16 min 100 % B, 16–17 min 100–15 % B, 17–20 min 15 % B. Mass spectrometric measurements were performed in negative ionization mode.

For derivatization of amino acids and biogenic amines, the supernatant was evaporated to dryness (SpeedVac, Eppendorf), resuspended in 50 µL of 5 % phenyl isothiocyanate (PITC) in ethanol: H_2_O: pyridine (1:1:1, v/v/v), and incubated for 25 min at RT. Subsequently, the samples were dried to remove excess PITC and resuspended in 10 µL 5 mM ammonium acetate in methanol. After incubation (10 min at 14,000 rpm) 90 µL of H_2_O: ACN + 0.2 % formic acid were added.

Prior to measurement, each derivative was diluted 1:25, and 10 µL were injected onto a Waters Acquity UPLC system coupled on-line with a QTRAP® 5000 mass spectrometer (Sciex, Framingham, USA). Chromatographic separation was achieved with an Agilent Zorbax Eclipse XDB-C18 column (3.5 µm, 3.0 x 100 mm) using a constant flowrate of 0.5 ml/min and water + 0.2 % formic acid, and ACN + 0.2 % formic acid as mobile phases A and B, respectively. The linear LC gradient was as follows: 0–0.5 min at 0 % B, 0.5–4 min 0–70 % B, 4–5.3 min 70 % B, 5.3–5.4 min 70–0 % B, 5.4–7.3 min 0 % B and the QTRAP was set up to positive ionization mode.

Under the same conditions, all undiluted samples were measured on an LC-MS/MS system using an Agilent 1290 II infinity UPLC system (Agilent Technologies Inc., Santa Clara, CA, USA) coupled with a QTRAP6500+® mass spectrometer (Sciex, Framingham, USA).

For identification and quantitation, a scheduled multiple reaction monitoring (MRM) method was used for both amino acids and SCFA, with specific transitions for every metabolite. External calibration curves were measured using linear regression. The peak areas of all samples and standards for linear regression were determined using SciexOS® software (v3.0.0, Sciex).

### (Meta-)proteomic measurements

To determine microbiota composition and functional pathway annotation for the microbiome, honeybee guts and brains, we used a (meta-)proteomics approach. All samples underwent lysis, protein clean-up, and proteolysis. For the lysis, screw-on Eppendorf tubes (Eppendorf, Germany) received 5–6 medium-sized glass beads (1 mm, Carl Roth) and three scoops of zirconia beads (0.1 mm, Carl Roth) before the individual guts and brains were added. Each sample received 200 μL of Triton X-100 lysis buffer (150 mM NaCl, 0.1 % SDS, 0.5 % Sodium Deoxycholate, 50 mM Tris HCl, 1 % Triton X-100, pH 7.4, with 1:25 protease inhibitor (cOmplete™, Roche)), followed by agitation in a FastPrep machine at 5.5 m/s for three 60-second rounds (MP Biomedicals™ FastPrep™-24, ThermoFisher Scientific™, Waltham, MA, USA). Samples were then incubated on ice for 60 min, with periodic mixing on a vortex device (every 10 min). Samples were then sonicated for 1 min at 30 °C, before centrifugation at 15,000 × g for 15 min (Centrifuge 5430 R, Eppendorf, Hamburg, Germany). The resultant protein concentration in the supernatant was quantified using the DC Protein Assay Kit (Bio-Rad, Hercules, CA, USA), according to the manufacturer’s instructions.

For protein clean-up and proteolysis, we used an adapted Single-Pot Solid-Phase-enhanced Sample Preparation (SP3) protocol based on Hughes et al. [24]. Briefly, lysate containing 100 µg of protein for the guts (30 µg for brains) were added to 1.5 mL Eppendorf tubes (Eppendorf, Germany) and adjusted to 200 µL with 100 mM Triethylammonium bicarbonate buffer (TEAB). The samples were then reduced (5 µL of 200 mM Tris(2-carboxyethyl) phosphine hydro-chlorine (TCEP) in 100 mM TEAB, with a 1-hour incubation at 55 °C and 300 rpm) and alkylated (5 µL of 375 mM Iodoacetamide (IAA) in 100 mM TEAB, incubated in the dark for 30 min at room temperature and 600 rpm). Then, 280 µL of ACN (100 %, unless otherwise stated) were added. SpeedBead Magnetic Beads (SpeedBead Magnetic Carboxylate, Cytiva, Marlborough, MA, USA) underwent two sets of washing in ddH_2_0 before the reduced and alkylated samples were added (10 µL beads for guts, 5 µL for brains) and the mixture was incubated for 10 min at room temperature. After 2 min of incubation on the magnetic rack, the supernatant was discarded, and the beads were rinsed twice with 200 µL of ethanol (70 %) and once with ACN while on a magnetic rack. Each rinsing step was followed by another incubation of 30 secs. All supernatants were removed and the beads were left to fully dry. We added 2 µL of Trypsin/Lys-c mix (Promega, Madison, WI, USA) for the guts (0.4 µL for brains) adjusted to 7 µL (5 µL for brains) of a 100 mM TEAB solution to the dried beads to initiate proteolysis. The samples were then incubated for 16 h at 37 °C. To stop proteolysis, 350 µL of ACN were added to the sample and incubated for 12 min at room temperature. Subsequent clean-up of the peptides involved discarding the supernatant and washing with 200 µL of ACN on the magnetic rack. Next, the beads were homogenized in 50 µL of 2 % (v/v) DMSO solution and sonicated for 1 min before 2 min incubation on the rack. The supernatant containing eluted peptides was transferred to a new Eppendorf tube. The DMSO step was repeated with pipette mixing instead of sonication, and the second part of the eluted peptides was recovered, resulting in a total volume of 100 µL containing 100 µg of protein (for the guts, 30 µg for the brains). Samples were dried in a SpeedVac, re-suspended in 0.1 % formic acid (FA) in LC/MS-MS water (100 µL for guts, 30 µL for brains) and centrifuged at 20,000 × g for 10 min. Subsequently, 50 µL (for the guts, 20 µL for brains) were transferred to LC-MS vials at a final concentration of 1 µg/µL and frozen at −80 °C until measurement.

For each sample, 1 µg of peptides was injected and measured on a Vanquish Neo nanoHPLC (ThermoFisher Scientific™, Waltham, MA, USA) with a C18-reverse phase trapping column (Acclaim PepMapTM 100, 75 μm × 2 cm, particle size 3 μm, nanoViper, ThermoFisher Scientific™, Waltham, MA, USA), followed by a C18-reverse phase analytical column (Double nanoViper™ PepMap™ Neo, 75 μm × 150 mm, particle size 2 μm, ThermoFisher Scientific™, Waltham, MA, USA). The LC separation operated on a 162 min long two-step gradient using mobile phases A (0.01 % FA in LC/MS H_2_O) and B (80 % ACN in LC/MS H_2_O with 0.01 % FA). Mobile phase B was increased from 4 % to 30 % in the first 100 min, followed by an increase to 55 % in the next 40 min. For the next 15 min, mobile phase B was increased to 99 %, which remained at until the end of the measurement. The flow rate for the entire separation was 300 nL/min.

Positive ionisation in occurred using a Nanospray Flex™ Ion Source (ThermoFisher Scientific™, Waltham, MA, USA) and were then detected using an Orbitrap Exploris™ 480 mass spectrometer (ThermoFisher Scientific™, Waltham, MA, USA) operating in data-independent acquisition (DIA) mode. The range for the MS1 scan was 350–1,500 *m*/*z*, with a resolution of 120,000. The normalised automatic gain control (AGC) target was set at 300 %, and we used a centroid data type. We used the automatic DIA window type, which comprised an isolation window of 24 *m*/*z* with a window overlap of 1 *m*/*z*, resulting in 48 scan events (Table S1).

### Database creation and protein assignment for DIA data analysis

We established a comprehensive proteome database for the honey bee and the bacterial species found in the worker gut. Uniprot (www.uniprot.org, v. 2024_02) were used to download proteomes (reference proteomes if possible) for all species, which were later separated into an *Apis mellifera* database and an *Apis mellifera +* bacterial species database. The database for the microbiota contained 16 species that were chosen based on recent literature of honey bee gut microbiome sequencing, grouped into their main phylotypes [13,[25], [26], [27]] (Table S2).

Mass spectrometric data processing was performed using Proteome Discvoverer (v.3.2, Thermo Fisher Scientific, San Jose, USA) with the CHIMERYS search engine. Search settings were set to trypsin (full), max. missed cleavage sites 2, and a max fragment mass tolerance 20 ppm. Carbamidomethylation of cysteine was set as a fixed modification. Proteins were considered as identified when at least one unique peptide was found and if the overall protein FDR (false discovery rate) was ≤ 0.01. Brain samples were searched against the *Apis mellifera* database, while the guts were searched against the *Apis mellifera +* bacterial species database.

### Structural and functional annotation of DIA data

The identified protein sequences were submitted to GhostKoala (https://www.kegg.jp/ghostkoala/), where KEGG orthologs were annotated to the proteins. With the data output from Proteome Discoverer (v3.2) and the NCBI reference database, an in-house written *R* script was utilised to calculate the relative abundances of each microbiota species in each sample and age group. The relative abundance was calculated by dividing the abundance of the species by the summed abundance of all species in the sample. Moreover, the script was used to calculate the relative intensities of different KEGG pathways in the microbiota, and for the honeybee guts and brains for each sample and age group. A filter of a minimum of five proteins per pathway and a minimum pathway coverage of 10 % was employed. Pathways were then filtered for known KEGG pathways in *Apis mellifera* and the relevant bacterial species for the microbiota (https://www.kegg.jp/).

Using this approach, we identified protein groups across the different age groups and studied matrices (Table S3). We detected 279 unique protein groups for the microbiota, with an average of 76 protein groups per sample. Of these, 68 % could be assigned to the four detected KEGG pathways. We identified 1,253 unique proteins for the honey bee gut with an average of 661 protein groups per sample. We could assign 77 % of these to KEGG pathways and found 59 distinct KEGG pathways. For the brain, 3,291 unique protein groups were found, with each sample having an average of 2,966 protein groups. We assigned 80 % to KEGG pathways, resulting in 104 distinct KEGG pathways being found.

Honey bee gut and brain pathways were then further filtered by significance (p < 0.05) of at least one age group in comparison to Day 0 and a log_2_ fold change (log_2_FC) of at least |1| (guts) and |0.5| (brains) of at least one age group in comparison to Day 0. This left 40 pathways for the gut (Fig. S9) and 42 pathways for the brain (Fig. S8). The top 18 for guts and top 24 pathways for brains were further filtered based on the condition that at least three of the age groups showed the above-mentioned log_2_FC in comparison to Day 0.

### Bioinformatic data analysis

All statistical analyses were conducted in *R* (v.4.3.1) and the threshold for significance was always p < 0.05 (two-tailed). Alpha diversity for bacterial abundance across age groups was calculated using the Shannon Index and beta diversity was analysed by computing a Principal Coordinates Analysis (PCoA) based on Bray-Curtis dissimilarity matrices. Taxonomic, metabolomic and functional profiles were analysed using principal component analysis (PCA) with subsets for all age groups, the establishment phase (Day 0 to Day 5) and the worker phase (In-hive worker, new and experienced forager). Significance was determined with a subsequent permutational multivariate analysis of variance (PERMANOVA) using the *vegan* package. Group differences were calculated using a Kruskal Wallis test due to the non-parametric nature of the data, followed by a Dunn’s pairwise comparison test with a Benjamini-Hochberg p-value correction. Log_2_FC were calculated in comparison to Day 0, after the data underwent a median-median normalisation. For better visualisation in the heatmaps, we used a k-means clustering algorithm to group pathways and metabolites that show the same trend across age groups. In accordance with the non-parametric data distribution, a Spearman’s correlation was utilised to analyse the correlation between amino acid and biogenic amine levels in the gut and brain, and the relative bacterial abundances and amino acid and biogenic amine levels in the gut. Relevant p-values were computed using algorithm AS 89 or via asymptotic *t* approximation in the *stats* package. All visualisation was done in R using the *ggplot2* package, and heatmaps were created using GraphPad Prism (v10.2.3, GraphPad Software, San Diego, CA, USA).

The capability of bacterial species listed in Table S2 to produce specific metabolites was predicted using metabolic modelling. Therefore, genome-scale metabolic networks were reconstructed using gapseq (development v.1.3.1 faf0f84 [28]) based on the protein sequences of the respective UniProt proteomes (Table S2). The reconstruction workflow consisted of five steps: (i) Reaction and pathway prediction, (ii) prediction of metabolite cross-membrane transporters, (iii) reconstruction of a draft metabolic network based on the results of *i* and *ii*, (iv) estimation of an organism-specific growth medium for subsequent (v) gap-filling of the metabolic network to enable biomass production. The final models were tested for their capability to produce those metabolites, which were also quantified in the metabolomic assessment of this study (see above for details). To this end, a sink reaction for the respective cytosolic metabolite (e.g. tyramine) was added to the model with a lower bound of 0 and an unconstrained upper bound. Flux balance analysis was applied to the modified model with the objective function set to the flux through the new sink reaction. If the optimal solution yielded an objective flux greater than zero, the model was considered capable of producing the respective compound. The simplex algorithm implemented in the IBM ILOG CPLEX Optimization Studio (v12.10, IBM, Armonk, NY, USA) was used as an LP solver.

## Results

### *Bombilactobacillus* and *Lactobacillus* spp. Make up the majority of the microbiome in in-hive bees from day 3

**Fig. 1:**
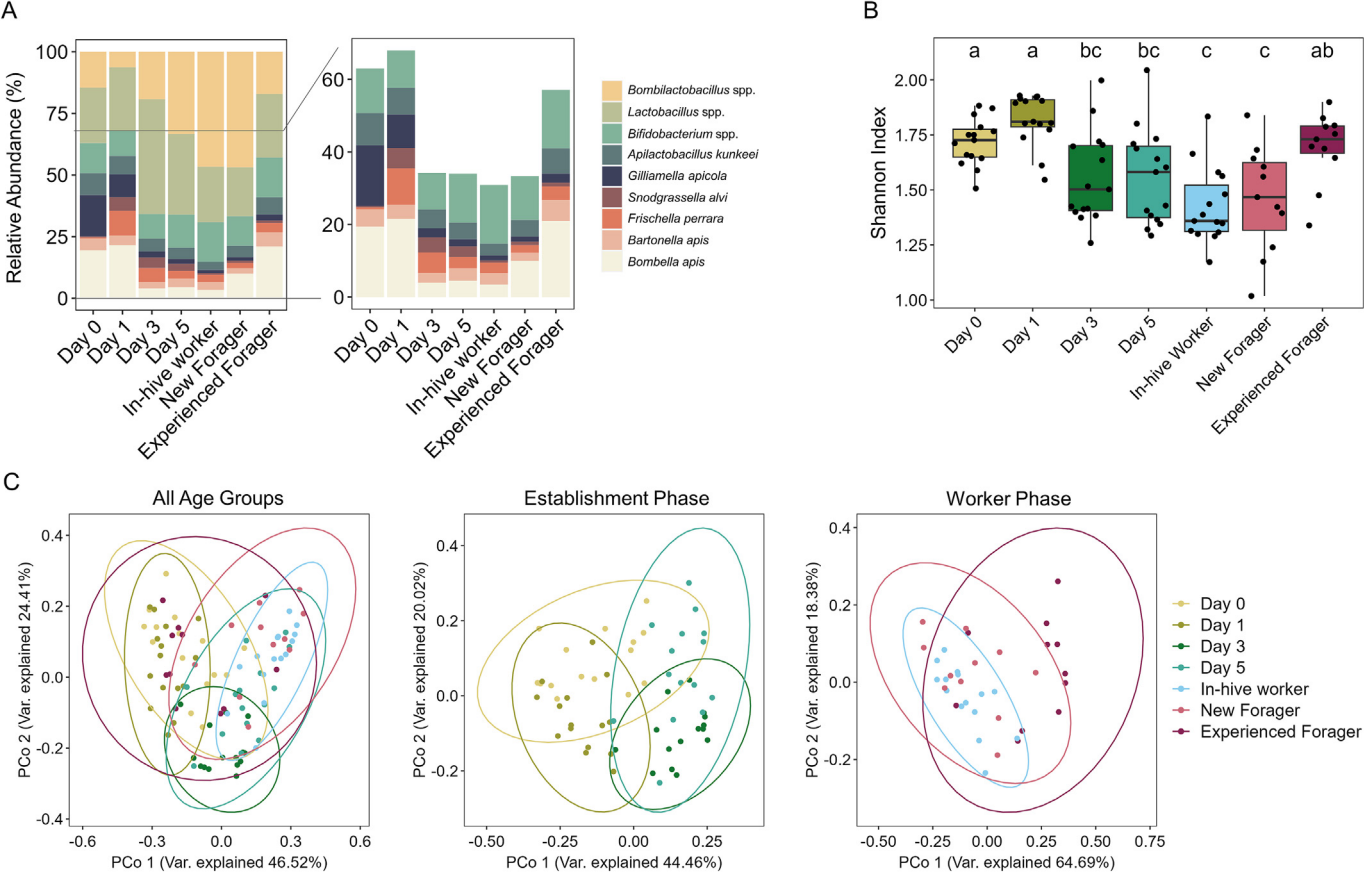
Metaproteomic-based analyses of bacterial abundances in honey bee microbiome across age groups. (A) Relative abundances of detected phylotypes across the different age groups. (B) Shannon Index of bacterial communities across age groups. (C) Principal Coordinate Analysis (PCoA) based on Bray-Curtis dissimilarity for all age groups, the establishment phase (Day 0 to Day 5) and worker phase (in-hive worker, new and experienced forager).

The composition of the gut microbiome and number of protein groups identified changed significantly across age groups. Protein numbers increased significantly from Day 3 onwards, as the microbiome established (Fig. S1, Table S3). While all phylotypes were detected in all age groups, there was significant variation in the relative abundances across the stages (Fig. 1A, Fig. S1). *Bombilactobacillus* spp. and *Lactobacillus* spp. made up the majority of the composition from Day 3 to new foragers, aligning with the decrease in α-diversity during for these age groups (Fig. 1B). *Bombilactobacillus* spp. increased from Day 1 to their maximum peak relative abundance in in-hive worker (46.7 %) and new foragers (46.9 %), followed by a significant decrease in experienced foragers. *Lactobacillus* spp. show an inverse pattern to this, with a maximum relative abundance on Day 3 (46.6 %), with a subsequent decrease until the new forager stage (19.7 %). *Gilliamella apicola* and *Bombella apis* both showed a significant reduction in their relative abundance from Day 3. While *G. apicola* relative abundance remained low across all following age groups, *Bombella apis* increased again significantly for experienced foragers. *Snodgrassella alvi* and *Bartonella apis* showed no significant changes in relative abundance across any age groups (Fig. S2). To understand how (dis-)similar communities between age groups were, we conducted a β-diversity calculation based on Bray-Curtis dissimilarity distances, plotted using a Principal Coordinates Analysis (PCoA). We observed overlapping clusters across age groups for microbial communities, implying similar communities (β-diversity, Fig. 1C). Days 0 and 1 show some differentiation from Days and 5, whereas samples belonging to the worker stages were mostly overlapping, with experienced foragers showing the greatest variability.

### Short-chain fatty acid profile changes significantly during the establishment phase, showing a clear distinction between days 0 and 1, and days 3 and 5

**Fig. 2:**
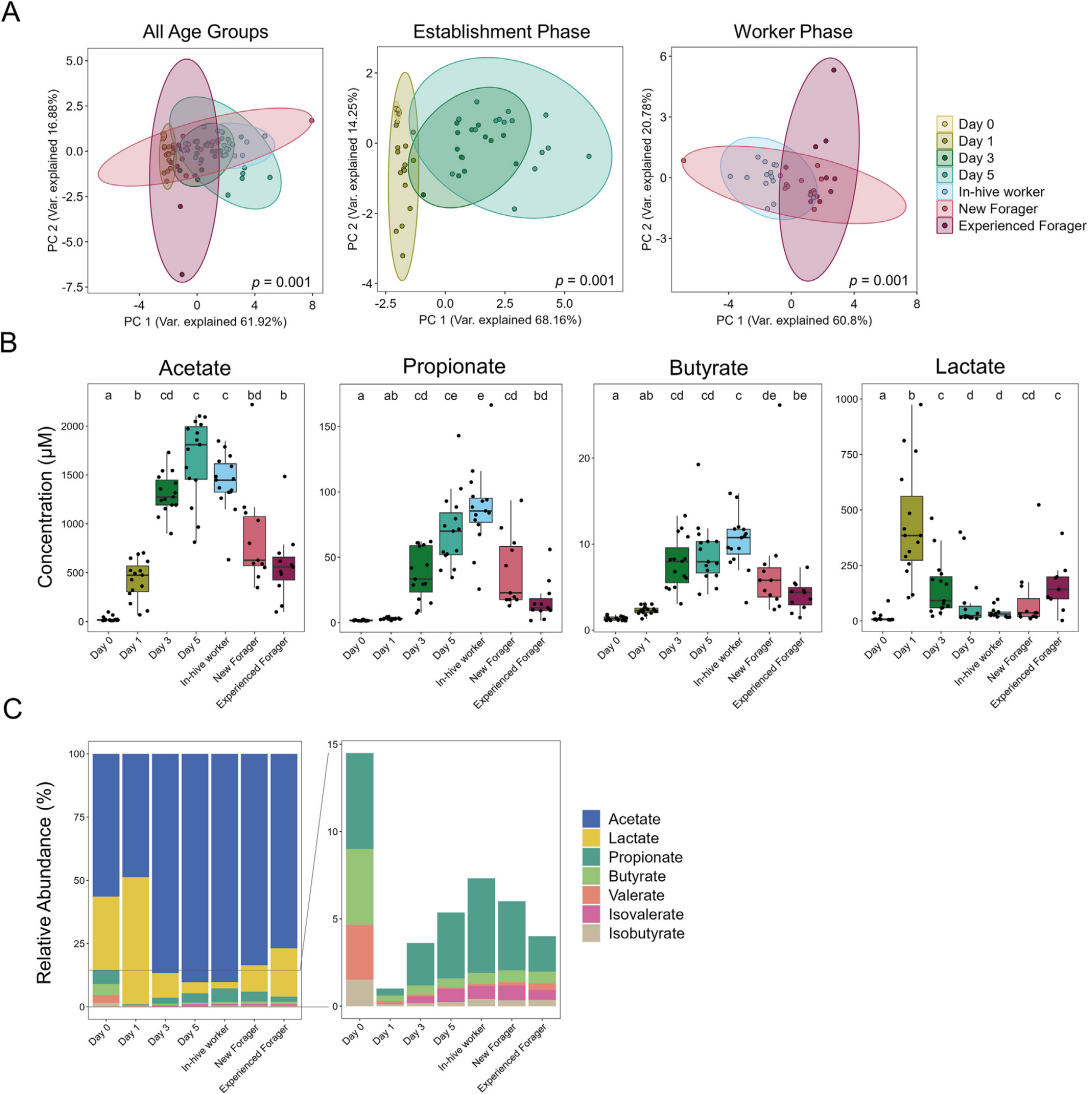
Metabolomic-based analyses of short-chain fatty acid (SCFA) concentrations in the honey bee guts across age groups (A) Principal Components Analysis (PCA) of all measured SCFA concentrations for all age groups, and establishment and worker phase. Significant differences (PERMANOVA) between age groups is displayed in the bottom right corner of each plot. The most distinct changes can be seen between Day 0 and Day 1 in comparison to Days 3 and 5, implying a major shift in the SCFA concentrations during the microbiome establishment phase. Day 0 shows the lowest rate of variability along the two principal components, while new and experienced foragers show one to two outliers each, thereby increasing the variability of these age groups. (B) Absolute concentrations of the main SCFA and lactate across all age groups. (C) Relative abundances of all measured SCFA and lactate across all age groups.

Short-chain fatty acids (SCFA) are an indicator of bacterial metabolism, and during the establishment phase, we can see a distinct change in the distribution of samples along the two principle components from Day 0 to Day 3 (Fig. 2A), indicating a shift in the overall SCFA profile during this time. The main SCFA, acetate, propionate and butyrate, make up the majority of the SCFA in all age groups and show a bell-shaped trend, with their highest concentrations occurring on Day 3, Day 5 and for in-hive workers. (Fig. 2B, C). Lactate concentrations were also measured and followed an inverse bell shape, with a maximum concentration on Day 1. Concentrations then decreased before significantly increasing in experienced foragers (Fig. 2B). Absolute concentrations for all six measured SCFA and lactate can be seen in Fig. S3.

### Amino acid levels indicate a correlation between brain and gut

We measured 21 amino acids in the brain and 20 (same as in the brain except for proline) in the gut. Additionally, the concentrations of six biogenic amines were measured in both body compartments.

**Fig. 3:**
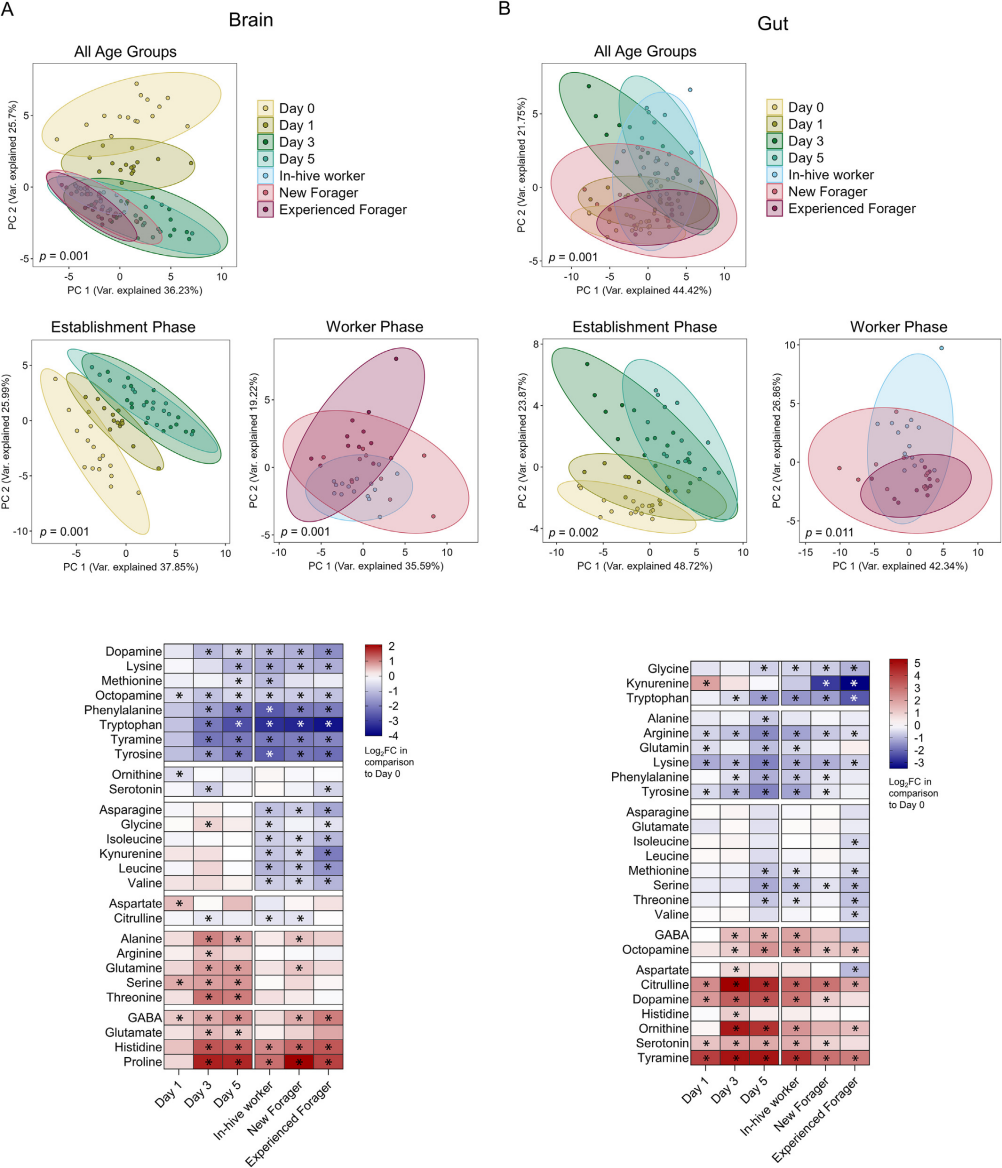
Metabolomic-based analyses of amino acid and biogenic amine levels in the honey bee brain and guts across age groups. PCA and log_2_FC of all measured amino acid and biogenic amine concentrations in the honey bee **(A)** brains and **(B)** guts for all age groups, and establishment and worker phase. Significant differences in the PCA (PERMANOVA) between age groups is displayed in the bottom right corner of each plot. Log_2_FC of each age group is shown in comparison to Day 0. Metabolites were grouped into clusters based on k-means clustering, meaning that amino acids that follow the same patter across age groups end up in the same cluster. Significant differences in concentrations (*p* < 0.05, Kruskal Wallis followed by Dunn’s test for multiple pairwise comparison) in comparison to Day 0 are shown with an asterisk. (A) PCA shows distinct ellipses for amino acid distribution on Day 0 and 1. During the worker phase, in-hive workers and both stages of foragers largely overlap, though variability is larger for new and experienced foragers. When looking at individual amino acids, the top three clusters show amino acids that significantly decrease in comparison to Day 0. The fourth cluster shows some variation between increases and decreases in concentration in comparison to Day 0, while the last two clusters contain amino acids that increase in concentrations as the bee age. Absolute concentration can be found in Supplementary Fig. S5 and S6. (B) In the gut, the PCA shows that Days 0 and 1 are not as distinct as in the brain. While Days 3 and 5 show different distributions to Days 0 and 1 during the establishment phase, the age groups in the worker phase do not differ significantly from one another (*p* = 0.011). Instead, they seem to overlap with the ellipses from Days 0 and 1. The clustering of log_2_FC of individual amino acids revealed that the metabolites shown in the top three clusters decrease significantly in one or more age groups in comparison to Day 0, while the bottom two clusters show amino acids that increase in several age groups. Absolute concentration can be found in Supplementary Fig. S7 and S8.

Amino acids and biogenic amines were grouped into clusters depending on their log_2_FC concentration changes in comparison to Day 0, to detect amino acids and biogenic amines that showed similar trends throughout development (Fig. 3). Brain tryptophan showed a significant decrease from Day 3 onwards, with the most considerable decrease in the worker phase (Fig. 3A, S5). Kynurenine, a metabolite of tryptophan, also showed significant reductions in all worker phases (Fig. 3A, S6). Serotonin is also a downstream product of tryptophan, but unlike tryptophan, it only showed substantial decreases on Day 1 and for experienced foragers (Fig. 3A). Similarly, to the brain, gut tryptophan and its metabolite kynurenine were part of the same cluster, with tryptophan showing a significant decrease from Day 3. In contrast, gut kynurenine showed a substantial drop during the foraging stages. Serotonin levels in the gut, on the other hand, showed a significant increase for all age groups except experienced foragers (Fig. 3B, S8).

For amino acids and biogenic amines involved in octopamine biosynthesis, we observed different trends in gut and brain. In the brain, tyrosine and tyramine were part of the same cluster, showing significant decreases from Day 3 onwards (Day 1 for octopamine, Fig. 3A). In the gut, however, tyramine and octopamine showed significant increases in concentrations (octopamine for all age groups apart from Day 1; tyramine for all age groups), while their precursor tyrosine showed significantly decreased concentrations for all age groups except experienced foragers (Fig. 3B).

**Fig. 4:**
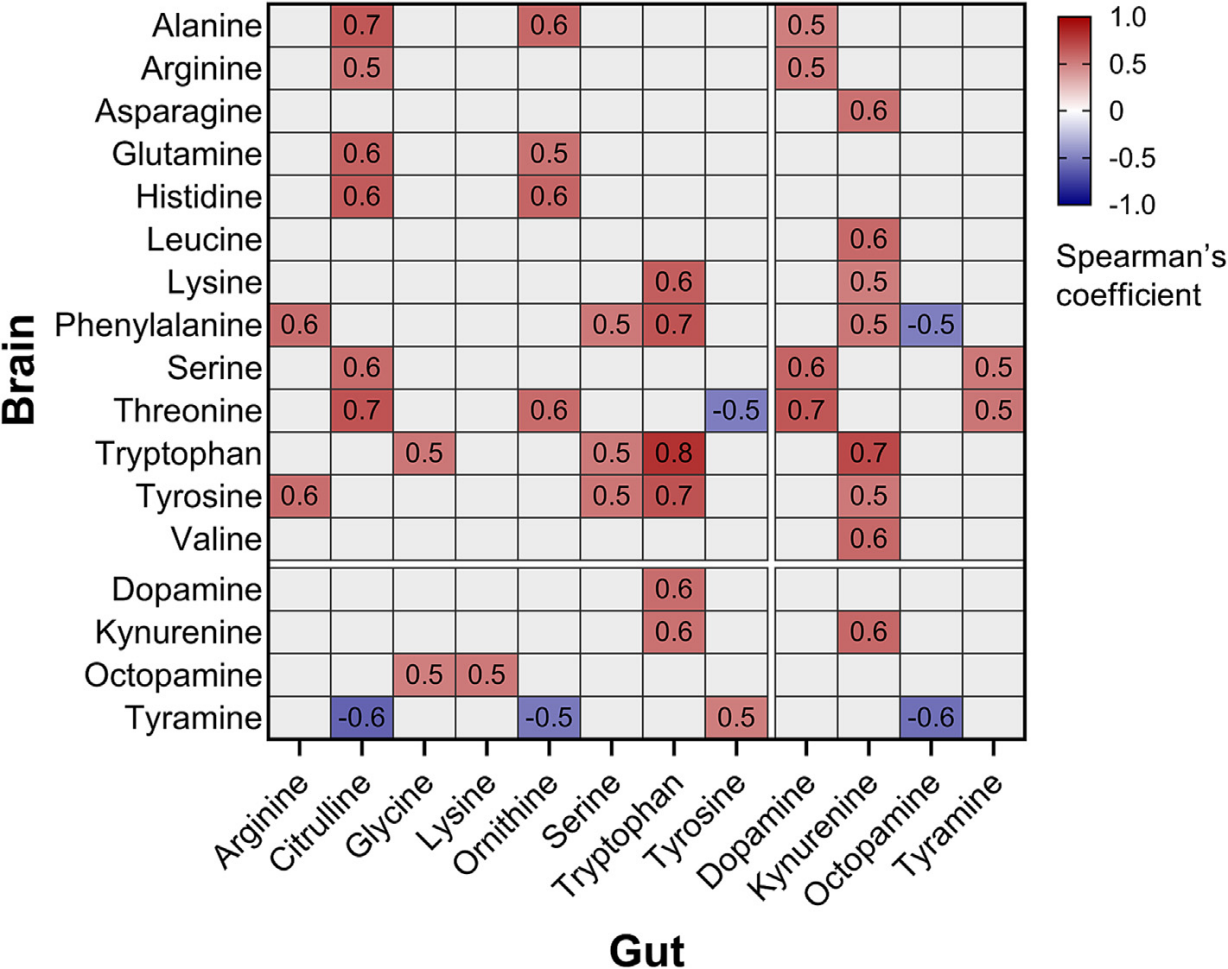
Correlation analysis of amino acid and biogenic amine concentrations between honey bee brain and gut. Subset of significant correlations (p < 0.05) between amino acid and biogenic amine levels between honey bee and gut, with a Spearman’s coefficient ρ >|0.5|. All significant correlations can be seen in Fig. S4.

To investigate whether certain concentrations of amino acids and biogenic amines followed the same trend between the gut and brain, we calculated the Spearman’s coefficient between concentrations and focused on statistically significant correlations (Fig. S4). Correlations with a Spearman’s coefficient |ρ| >0.5 are shown in Fig. 4. Tryptophan levels in the brain and gut were positively correlated (ρ = 0.8), as well as tryptophan levels in the brain with kynurenine levels in the gut (ρ = 0.7). Tyrosine is metabolised into tyramine in the octopamine pathway. Tyrosine concentrations in the gut were positively correlated to tyramine concentrations in the brain (ρ = 0.5). In contrast, tyrosine levels in the brain were negatively correlated to tyramine levels in the gut (ρ = -0.2, Fig. S4). Together with the finding that high tyramine levels in the brain are negatively correlated with tyramine (ρ = -0.4, Fig. S4) and octopamine (ρ = -0.5) concentrations in the gut, it could suggest some relationship between tyrosine and tyramine biosynthesis or transport between the two body parts.

Absolute concentrations for all amino acids and biogenic amines in the brain can be seen in Fig. S5 and S6, and in the gut in Fig. S7 and S8.

### Functional pathways in the brain, gut, and bacterial proteins

**Fig. 5:**
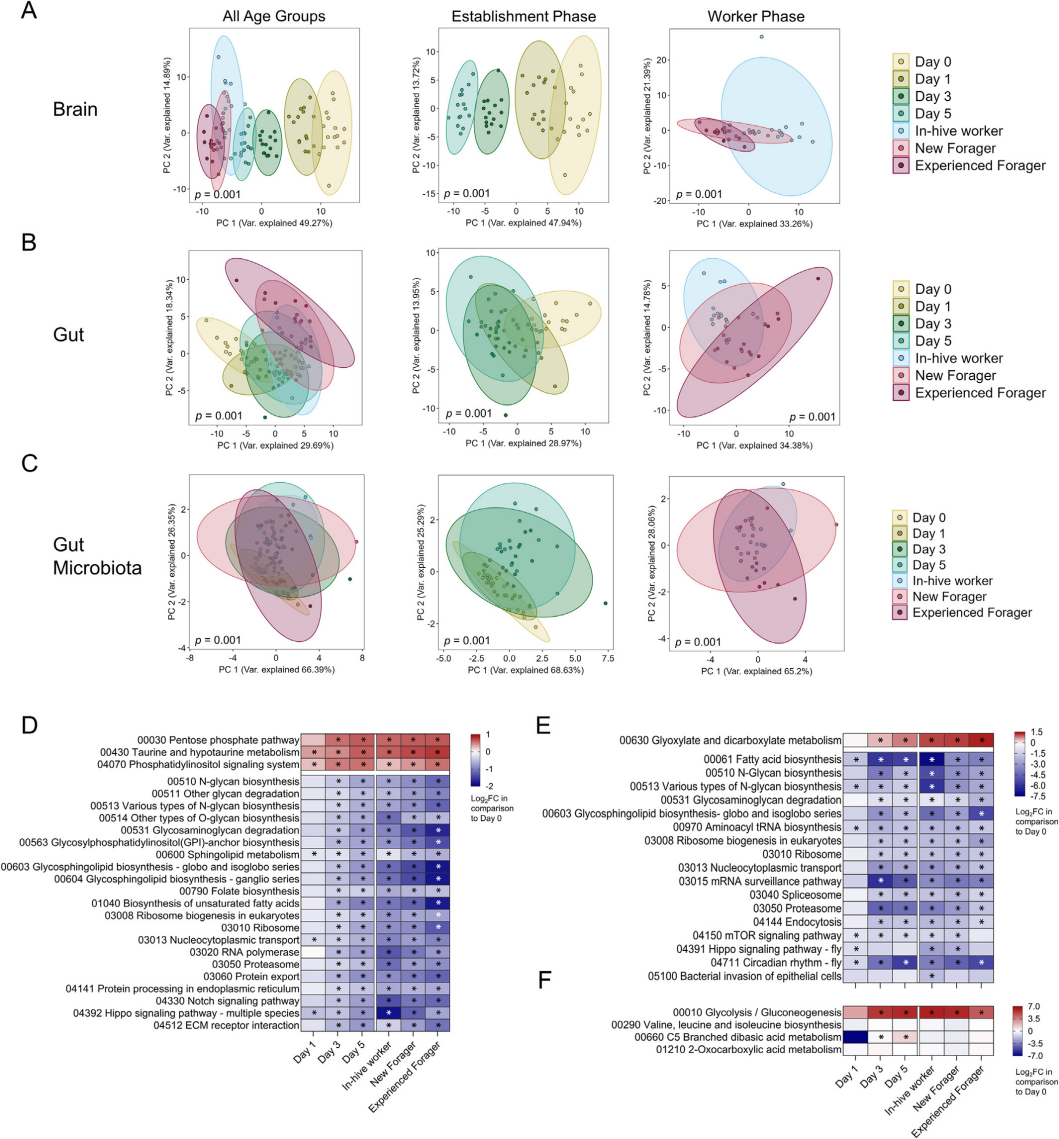
Proteomic-based analyses of KEGG pathways in honey bee brain, guts and bacterial community across age groups. PCA of all detected KEGG pathways in the **(A)** honey bee brain, **(B)** honey bee gut and **(C)** bacterial community for all age groups, and establishment and worker phase. Significant differences (PERMANOVA) between age groups is displayed in the bottom right corner of each plot. Log_2_FC of the top KEGG pathways of each age group in comparison to Day 0 are shown on the right for each studied matrix. Pathways were grouped into clusters based on k-means clustering, meaning that pathways that follow the same patter across age groups end up in the same cluster. Significant differences in pathway abundances (*p* < 0.05, Kruskal Wallis followed by Dunn’s test for multiple pairwise comparison) in comparison to Day 0 are shown with an asterisk. (A) Pathways in the honeybee brain show a clear differentiation on the PCA plot during the establishment phase (*p* = 0.001), while workers with an established microbiome did not show significant differences along the two principle components (*p* = 0.033). The top 20 pathways were clustered into those that decrease and increase significantly in certain age groups in comparison to Day 0. (B) The PCA of all age groups in the honeybee gut shows a clear distinction of Day 0 to the other age groups, while differentiation can also be seen within the establishment and worker phase (*p* = 0.001 for all). The top 20 pathways shown are clustered in the same way as the ones in the brain. (C) Three functional pathways were identified in the gut microbiota community. We can see an increased variability in the distribution of workers from Days 3 and 5, and in-hive workers. Samples from Days 0 and 1, and new and experienced foragers, on the other hand, show very little variability. There is only one cluster for the individual log_2_FC of the KEGG pathways in comparison to Day 0, and all pathways increased significantly in at least one age group.

Especially during the establishment phase, there are distinct clusters for the brain KEGG pathways for each age group (Fig. 5A). Important energy metabolism pathways such as glycolysis/gluconeogenesis and the pentose phosphate pathway were significantly more abundant from Day 3 (Fig. S9). Glycans are essential for cell signalling, but most N-glycan biosynthesis pathways showed significantly lower abundances in all age groups except Day 1 (Fig. 5D). Pathways involved in protein transcription, processing and transport were also significantly less abundant from Day 3 (nucleocytoplasmic transport from Day 1).

In the gut, pathways related to protein transcription and transport showed significantly lower abundances from Day 3 (aminoacyl tRNA biosynthesis from Day 1, Fig. 5E). Also similar to the brain, pathways related to N-glycan biosynthesis were significantly decreased from Day 1 and Day 3, respectively. Glycolysis/gluconeogenesis had higher abundances in new and experienced foragers, in line with the transition from in-hive to foraging tasks (Fig. S10). The abundance of the citrate cycle/TCA cycle significantly increased from in-hive workers and remained high for both foraging stages (Fig. S10). Pathways related to drug and xenobiotic metabolism increased in their relative abundance in the transition from in-hive workers to new foragers, and then once again for experienced foragers compared to new foragers.

Four KEGG pathways were detected for the bacterial proteins (Fig. 5F). Days 0 and 1 showed the lowest variation in the PCA analysis, while the foraging stages displayed the largest. Glycolysis/gluconeogenesis was significantly upregulated from Day 3 onwards. The valine, leucine and isoleucine pathway, and the 2-Oxocarboxylic acid metabolism pathway did not significantly change across any age groups.

### Increasing concentrations of GABA in the brain of foragers align with increased pathway intensity and enzyme abundance

**Fig. 6:**
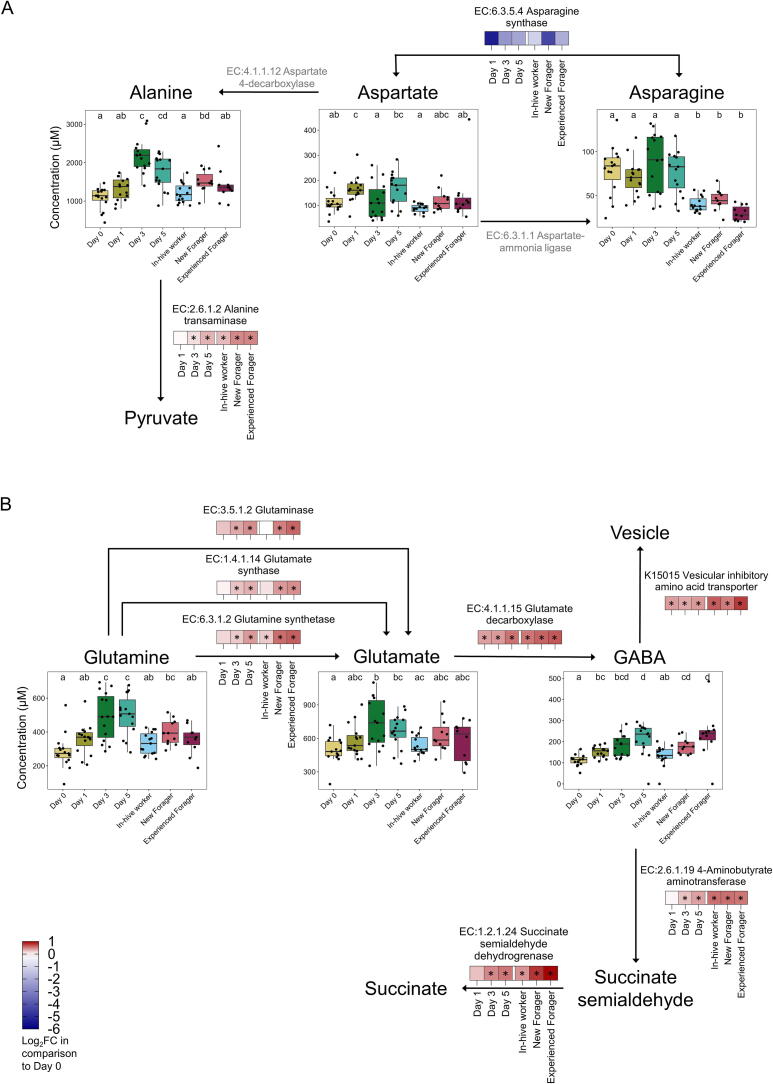
Simplified alanine, aspartate and glutamate metabolism (KO 00250) with abundances of enzymes in honey bee brain. For each enzyme, log_2_FC of each age group in comparison to Day 0 is shown for (A) alanine and aspartate metabolism and (B) glutamate metabolism. Significant differences in enzyme abundance (*p* < 0.05, Kruskal Wallis followed by Dunn’s test for multiple pairwise comparison) in comparison to Day 0 are shown with an asterisk. Absolute concentrations of amino acids and biogenic amines involved in the pathway are shown.

The alanine, aspartate and glutamate metabolism (KO 00250) in the honey bee brain increased significantly from Day 3 in comparison to Day 0, with a peak abundance in foragers (Fig. S9). To investigate whether these increased pathway abundances are related to amino acid and biogenic amine production, we integrated the metabolomic and proteomic data (Fig. 6). We quantified the abundance of alanine transaminase, which metabolises alanine into pyruvate, to be significantly increased from Day 3 onwards, with the highest increase in new (log_2_FC = 0.46) and experienced foragers (log_2_FC = 0.48). Enzymes that catalyse the conversions of aspartate to asparagine and vice versa were not detected or not significantly affected in any age group. In terms of glutamate metabolism, all three enzymes that can metabolise glutamine to glutamate (EC 3.5.1.2, EC 1.4.1.14, EC 6.3.1.2) are significantly upregulated on Day 3, in line with a significant increase in the glutamate concentration on the same day. However, all enzymes show the greatest upregulation during the forager stages, yet glutamate concentrations remain unchanged. (Fig. 6B). However, glutamate decarboxylase (EC 1.4.1.15), which metabolises glutamate into GABA, is upregulated in all age groups in comparison to Day 0, but shows its greatest log_2_FC during the forager stages (new: log_2_FC = 0.60; experienced: log_2_FC = 0.653, which is reflected by the increasing GABA concentrations during the forager stages in comparison to in-hive workers.

## Discussion

Honey bee worker development is characterised by behavioural task differentiation, which is accompanied by a range of molecular changes. To get greater insights into the role of the host, microbiota, and their interaction in this development, we employed a multi omics approach, based on (meta-)proteomics and metabolomics, to investigate the age-dependent shifts in the honey bee workers' microbiota, gut, and brain. We found that KEGG pathway abundances in all matrices showed either consistent increases or decreases in comparison to Day 0. Many SCFA, amino acids and biogenic amines fluctuated throughout development, often in association with specific behavioural or microbial changes. The establishment phase, characterised by the formation of the microbiota community, showed the most significant changes in all omics profiles. This implies a crucial role in their molecular and behavioural development for the first few days in a worker’s life.

### Structure and function of the microbiota community are associated with age and task of worker bees

The microbiota plays a crucial role in aiding digestion and nutrition, as it is able to break down compounds that the host could otherwise not utilise [[29], [30], [31]]. Although the bacteria act as a community and often complement each other in terms of function [32], certain phylotypes are associated with certain roles [15,30], which are reflected by the requirements of the host and bacterial abundance. For example, nurse bees ingest more pollen for glandular protein production, while foraging bees ingest only small amounts of pollen for their own metabolic needs [33]. As *Lactobacillus* spp. and *Bombilactobacillus* spp. are known for play a key role in breaking down pollen compounds and complex sugars [14], their significant decrease in relative abundances during the experienced forager stages in comparison to in-hive bees from Day 3 and Day 5, respectively, could be related to the decrease in pollen ingestion. Thus, task differentiation may be the factor driving shifts in the microbiota composition in this case. Frischella perrara colonizes the pylorus, the region connecting the ileum and the midgut, where it is known to strongly stimulate host immune responses [34]. Its significant increase from Day 1 may reflect the need for heightened immune activity as young worker bees begin encountering environmental microbes and potential pathogens [35]. The presence of F. perrara can trigger the production of antimicrobial peptides, which in turn help the host defend against these microbial challenges [34]. Independent from host interaction, *Bombella apis* can produce specific antifungal metabolites [36], which could cause the increase of its relative abundances in new and experienced foragers in comparison to in-hive workers, as foragers have been shown to harbour a more diverse and variable fungal community in their guts [37].

The microbiota seems to be tightly interlinked with their host and its requirements, which in the honeybee is often reflected in terms of task differentiation and immune responses. However, some of the strains are also known for producing short-chain fatty acids (SCFA) during carbohydrate fermentation. SCFA can act as an energy source for gut epithelial cells, affect pH and oxygen concentrations, and act as signalling molecules in immune responses [38,39]. Acetate was the main SCFA found in the honey bee gut, and *Gilliamella apicola* has been identified as one of its main producers [38,40]. *G. apicola* and *Snograssella alvi* form a biofilm in the ileum, where they engage in cross-feeding [15]. *G. apicola* relative abundance stabilises from Day 3, in line with the significant increase in acetate concentrations (Fig. S2 and Fig. 2B). While its role in honey bees is not yet fully understood, it is known to be absorbed by gut cells for energy metabolism of colonocytes [41], act as an antimicrobial agent and possibly have anti-inflammatory effects [42] in other model organisms.

It is known that many species can synthesise a range of amino acids, but their contribution to the synthesis of biogenic amines is still unclear [15,43]. Combining omics data with machine learning approaches led us to hypothesise that *Snodgrassella alvi* is able to synthesise the most amino acids and biogenic amines in the worker gut microbiota, with 23 out of 27 measured metabolites (Fig. S12), which aligns with previous findings [14]. No bacteria seemed able to synthesise dopamine, kynurenine, octopamine or serotonin (Fig. S12), implying that they can produce the precursors while the transformation to the biogenic amines occurs in the gut lumen.

Further research is needed to better understand the interaction of task differentiation and diet on microbiome composition and function. Our detection of approximately 80 bacterial protein groups per sample limited the functional insights obtained, allowing the identification of only four KEGG pathways with adequate minimum coverage. Separate analysis of intra- and extracellular metabolites would likely improve the understanding of the role of bacterial species and the host in the synthesis, transport and degradation of metabolites. *In vitro* colonisation of single strains or a community could be valuable for understanding bacterial function at the strain level, as they allow controlled assessments of specific microbial activities. However, these experiments do not replicate the complex interactions present in a host system, where environmental factors, microbial consortia, and host-specific influences play crucial roles in shaping functionality [44]. Instead, the systematic colonisation of workers with various species combinations, followed by validation under field conditions, could further reveal intra-microbiome and host-microbiome interactions.

### Amino acids and biogenic amines are involved in division of labour and their concentrations seem to be mediated partially by gut-brain interactions

Biogenic amines are neuroactive molecules that can act as neurotransmitters or neuromodulators and have been shown to play a critical role in the regulation of certain tasks [[45], [46], [47], [48]]. GABA, for example, is a key inhibitory neurotransmitter in vertebrates and invertebrates [49], is involved in learning and motor control in honey bees [[50], [51], [52]]. Unlike other studies [50], we found GABA concentrations to be highest in experienced foragers. However, by integrating the metabolomic and proteomic approach, we suggest that GABA was sequestered into vesicles, as indicated by the high abundance of the vesicular inhibitory amino acid transporter (soluble carrier family 32, K150515) during forager stages, meaning it was likely not active at the synapses. Moreover, high abundances of 4-aminobutyrate aminotransferase (EC: 2.6.1.19) and succinate semialdehyde dehydrogenase (EC: 1.2.1.24) in foragers suggest that more GABA is metabolised into succinate, which significantly contributes to mitochondrial respiration [53] and plays a crucial role in ATP production in the TCA cycle [54], crucial for the energy-intensive foraging stages. Whereas GABA in vertebrates is known to be transported across the blood–brain barrier [55], we found no correlation between GABA and/or glutamate levels in the gut and brain, implying that it is regulated locally in honey bees.

Tryptophan, on the other hand, is known to be transported between the gut lumen to the brain, where it is involved in learning in worker bees [56,57]. It is primarily obtained through diet, and is the sole precursor of the neurotransmitter serotonin [58], which is mainly found in the hindgut and plays a role in regulating gut contractions [59]. Alternatively, tryptophan can also be catabolised into indole derivates or kynurenine via the kynurenine pathway [58]. While Zhang, et al. [57] showed that *Lactobacillus* spp. colonisation can inhibit the kynurenine pathway by suppressing tryptophan dioxygenase [60], we found no significant negative correlation between *Lactobacillus* spp. and kynurenine levels (Fig. S11). Instead, we observed a significant positive correlation between brain and gut tryptophan concentrations (Fig. 4), implying that it is transported from the brain to the gut [10], where it is metabolised into serotonin. This could explain the significant negative correlation between brain tryptophan levels and gut serotonin levels (Fig. S4). In the brain, serotonin decreased significantly in experienced foragers, which improves foraging efficiency as foragers with high levels of the neurotransmitter showed decrease food intake and sucrose sensitivity [61].

Moreover, there seems to be some transport of the amino acids and biogenic amines involved in octopamine and dopamine biosynthesis. Both have shown to be largely involved in task differentiation and learning [45,47,62]. Based on the positive correlation of tyrosine between the gut and brain, we suggest that tyrosine is transported from the gut to the brain, where it is further utilised in biogenic amine production. In the gut, most tyrosine appears to be metabolised into tyramine, dopamine, and octopamine on Day 5 and in in-hive workers. However, during the foraging stage, tyrosine levels increase significantly compared to in-hive workers (Fig. S7), while tyramine and octopamine levels decrease, suggesting a reduction in this metabolic pathway. This shift may result from changes in *Lactobacillus* spp. abundance (Fig. S2), as these bacteria are known to express tyrosine decarboxylase [63], which catalyses the conversion of tyrosine to tyramine. *Lactobacillus* spp. abundance is highest on Days 3 and 5 and in in-hive workers, mirroring tyramine levels and reflected in their positive correlation. The decline in *Lactobacillus* spp. abundance may therefore reduce tyramine and octopamine synthesis, highlighting the microbiota’s role in amino acid biosynthesis while also demonstrating the challenges of interpreting trends across multiple omics datasets. Overall, regulating amino acid and biogenic amine levels in the gut and brain, along with metabolite transport between these tissues, appears to play a crucial role in modulating task-specific behaviours and division of labour.

### Potential impacts of microbial and metabolic shifts in behavioural maturation and division of labour

The observed correlations between microbial and metabolic shifts and behavioural modulation raise the important question of causality and directionality. For example, experienced foragers showed a significant decrease in the relative abundance of *Bombilactobacillus* spp. (Fig. S1). These bacteria are known to play a central role in pollen digestion [14], and their highest relative abundances coincides with Days 5 and in-hive workers, when pollen intake is highest. Their decline in experienced foragers, who ingest less pollen, suggests that the microbial shift was likely a consequence of the dietary shift rather than a driver of behavioural transition.

In contrast, the significant increase of *F. perrara* from Day 1 may result as a response to increasing pathogen loads [35], or in anticipation of the increased exposure, suggesting a preparatory, host-microbiome regulated role. To determine causality, experimental studies that expose young workers to different microbial pathogens and monitor resulting shifts in *F. perrara* abundances could be useful.

Moreover, the date implies some relationship between the concentration of metabolites such as SCFA and the division of labour. In mammals, acetate can regulate enzymes and act as signalling molecules [64]. We observed increased acetate levels from Day 1 onward (Fig. 2B), which may indicate a role in initiating juvenile hormone (JH) signalling. In mosquitoes, acetate exposure has been shown to stimulate JH III production even in the presence of JH inhibitors [65]. While we did not measure JH levels, the pattern supports a hypothesis that acetate could influence endocrine pathways involved in behavioural maturation in honey bees. The subsequent decline of acetate in foragers may reflect a reduced requirement once certain behavioural transitions have taken place.

Looking at other metabolites, amino acid and biogenic amine profiles suggest that serotonin may play an important role in the nurse-to-forager transition. High correlation between gut and brain tryptophan levels show the potential of gut-brain transport of this specific amino acid. Although brain serotonin concentrations remain relatively stable, gut serotonin increases significantly on Days 3, 5, and in in-hive workers. This is most likely caused by the conversion from tryptophan, which decreases throughout the same days. However, serotonin in new and experienced workers is significantly decreased (Fig. S8). Previous research in ants linked serotonin concentrations to decreased nectar feeding [66], and in honeybees spiking serotonin into the head of workers also decreased feeding behaviour [59]. Given the presence of serotonin receptors in the gut [59], it is plausible that decreased gut serotonin levels contribute to the transition to foraging. Notably, these changes are already evident in new foragers, suggesting that modulation of neurotransmitter levels may precede and help drive behavioural changes, rather than merely reflecting them.

## Conclusion

In conclusion, our study offers a detailed overview of changes in the microbiome, metabolomic, and proteomic profiles of honey bee workers across different life stages. To deepen our understanding the impact of various metabolites and pathways, as well as the gut-brain relationship, we recommend integrating omics analyses focusing on specific metabolic routes. This approach could clarify gut-bacterial interactions and gut-brain cross-communication. To understand the contribution of the microbiota community and individual species, more studies should focus on *in vitro* and *in situ* studies that investigate amino acid synthesis and microbiota-host interaction. Moreover, further research should explore differences between new and experienced foragers to determine how individual experience affects molecular processes and to establish links with age, learning and memory. Lastly, while our study identified some metabolic correlations between the gut and the brain, further *in situ* investigations are needed to validate these connections. Understanding gut-brain interactions in honey bees is crucial for elucidating the role of the microbiota in worker development.

## Ethics statement

No animals that are subject to ethics approval were used in this study. However, handling times and contact were minimised to decrease stress to the individuals.

## CRediT authorship contribution statement

**Cassandra Uthoff:** Conceptualization, Data curation, Formal analysis, Investigation, Methodology, Project administration, Resources, Visualization, Writing – original draft, Writing – review & editing. **Yelyzaveta Yakovlyeva:** Data curation, Formal analysis, Writing – original draft, Writing – review & editing. **Beatrice Engelmann:** Formal analysis, Data curation, Writing – review & editing. **Ulrike Rolle-Kampczyk:** Writing – review & editing. **Sven-Bastiaan Haange:** Data curation, Writing – review & editing. **Abdulrahim T. Alkassab:** Conceptualization, Methodology, Writing – review & editing. **Jens Pistorius:** Writing – review & editing. **Fynn Brix:** Formal analysis, Writing – review & editing. **Silvio Waschina:** Formal analysis, Writing – review & editing. **Andreas S. Thum:** Conceptualization, Supervision, Writing – review & editing. **Nico Jehmlich:** Conceptualization, Visualization, Supervision, Writing – original draft, Writing – review & editing. **Martin von Bergen:** Conceptualization, Supervision, Funding acquisition, Writing – review & editing.

## Funding

The funding for the corresponding author is provided by the Bundesministerium für Ernährung und Landwirtschaft (BMEL) as part of the Sens4Bee project with the funding number 281C306A19.

## Declaration of competing interest

The authors declare that they have no known competing financial interests or personal relationships that could have appeared to influence the work reported in this paper.

## Data Availability

The (meta-)proteomics mass spectrometry and Spectronaut SEARCH files are available on the ProteomeXchange consortium led by the PRIDE partner repository with the data set identifier PXD057060. Metabolomic data is available on Metabolomics Workbench with the study ID ID ST003552 and the https://doi.org/10.21228/M8SV5J.
