# Supplementary material for: Comprehension of the age-dependent gut and brain interaction of honey bee workers by integration of multi omics approaches
Source: J Adv Res. 2025 Jul 30;82:469–83. doi: 10.1016/j.jare.2025.07.045 (PMC13001044; doi:10.1016/j.jare.2025.07.045)
Supplement: Supplementary Data 2 [file mmc2.pdf]

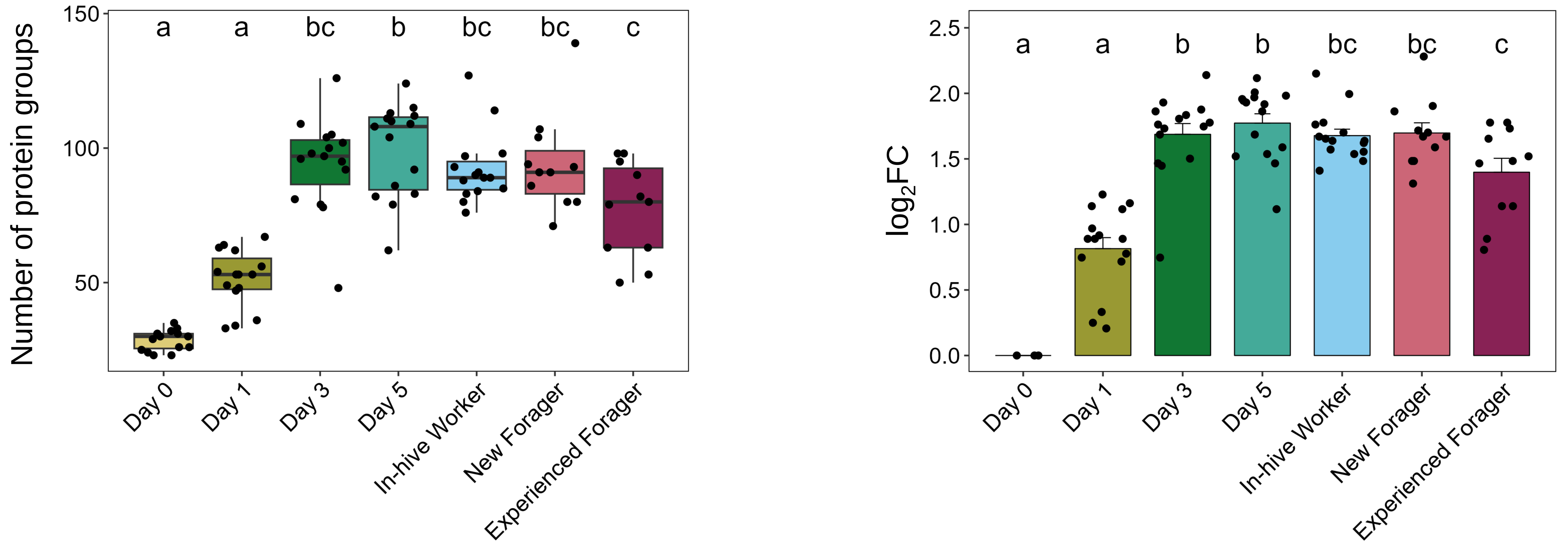

Supplementary Figure 1: Changes in the absolute number of detected protein groups of all bacterial phylotypes across all age groups and as a log_2 fold change (log_2FC) in comparison to Day 0. There are n = 15 samples for groups Day 0 to in-hive workers, and n = 11 for new and experienced foragers. Significant changes between age groups were calculated using a Kruskal Wallis test followed by pairwise comparison using a Dunn's test. *p*-values were adjusted using the Benjamini-Hochberg method. Significance is shown using the compact letter display.

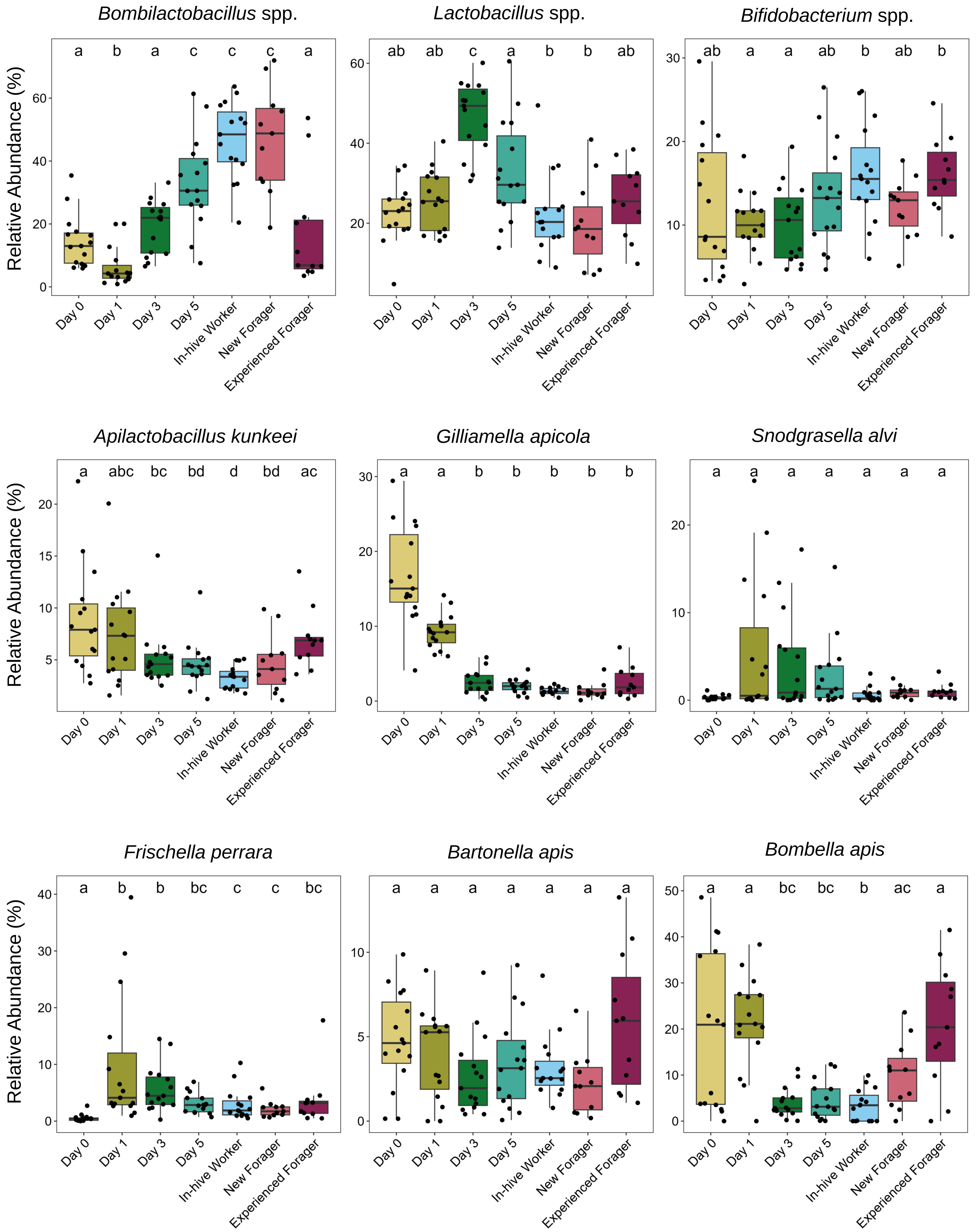

Supplementary Figure 2: Changes in the relative abundances of all bacterial phylotypes across all age groups. There are n = 15 samples for groups Day 0 to in-hive workers, and n = 11 for new and experienced foragers. Significant changes between age groups were calculated using a Kruskal Wallis test followed by pairwise comparison using a Dunn's test. *p*-values were adjusted using the Benjamini-Hochberg method. Significance is shown using the compact letter display.

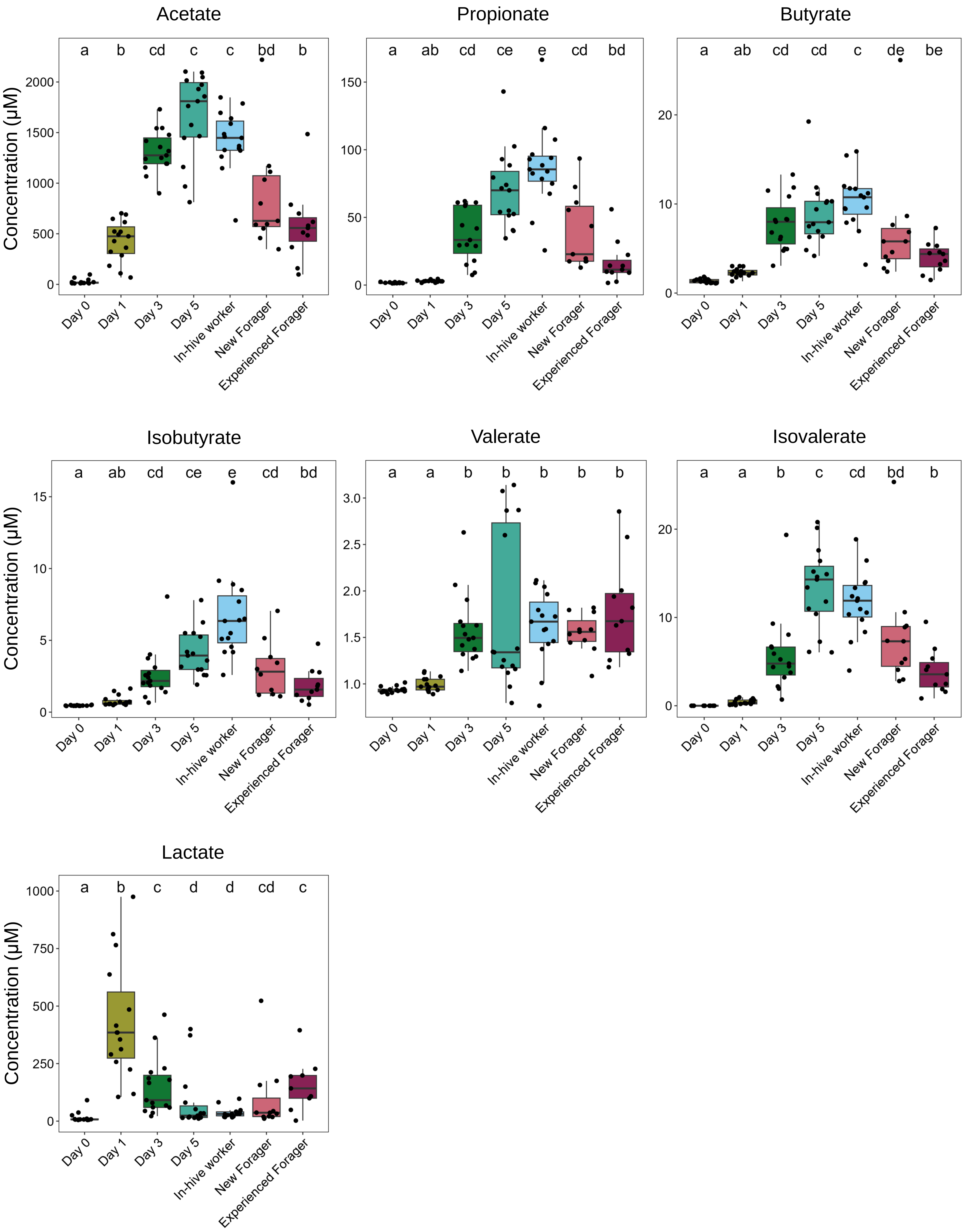

Supplementary Figure 3: Concentrations of all measured short-chain fatty acids (SCFA) and lactate in the honeybee guts across all age groups. There are n = 15 samples for groups Day 0 to in-hive workers, and n = 11 for new and experienced foragers. Significant changes between age groups were calculated using a Kruskal Wallis test followed by pairwise comparison using a Dunn's test. *p*-values were adjusted using the Benjamini-Hochberg method. Significance is shown using the compact letter display.

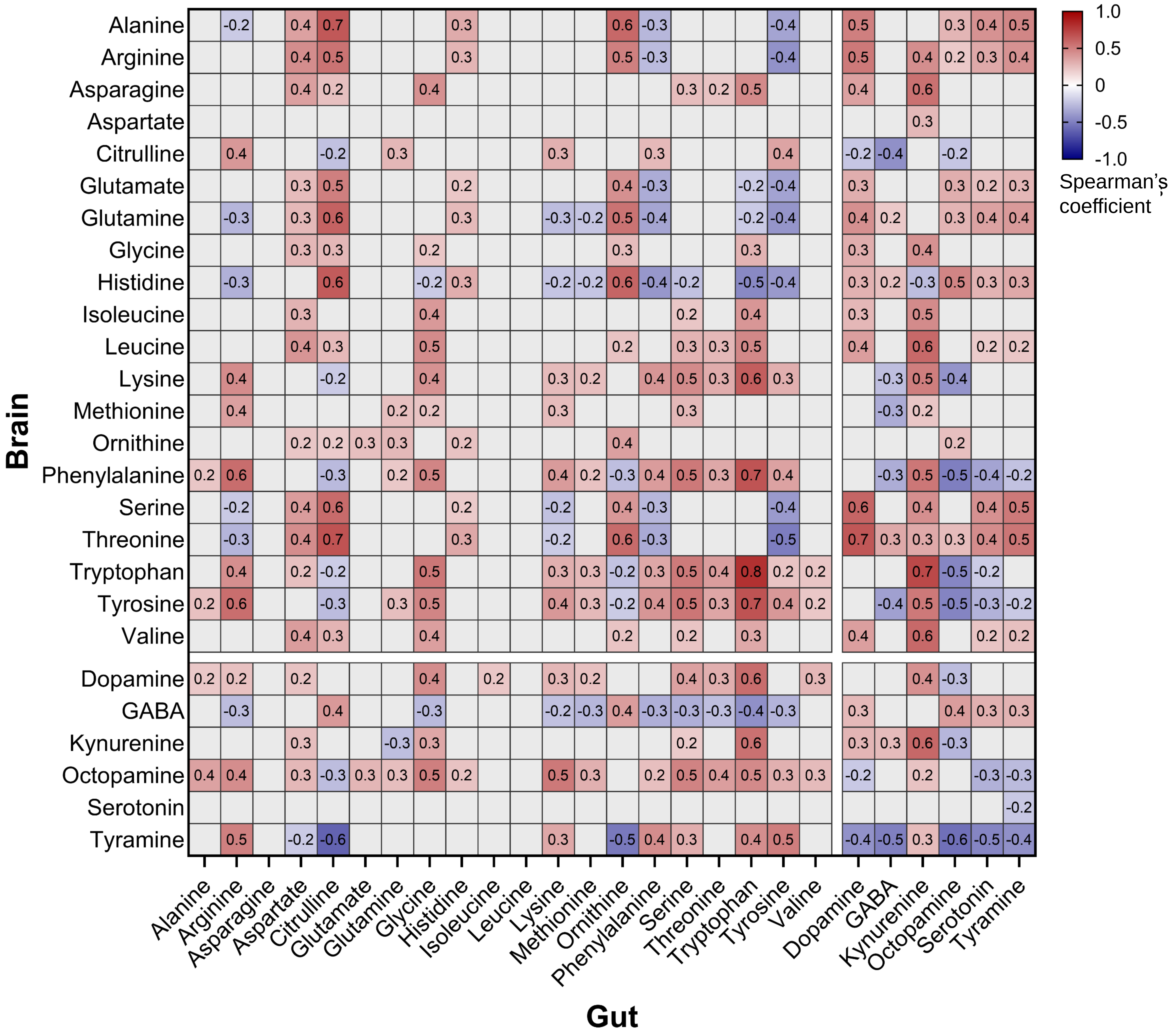

Supplementary Figure 4: Correlation analyses of amino acid and biogenic amine concentrations between gut and brain. Only significant correlations are shown. A Spearman's correlation was utilised and the relevant *p*-values were computed using algorithm AS 89 or via asymptotic *t* approximation in the *stats* package in *R*. There are n = 15 samples for groups Day 0 to in-hive workers, and n = 11 for new and experienced foragers and the analysis is based on data from single samples (n = 97).

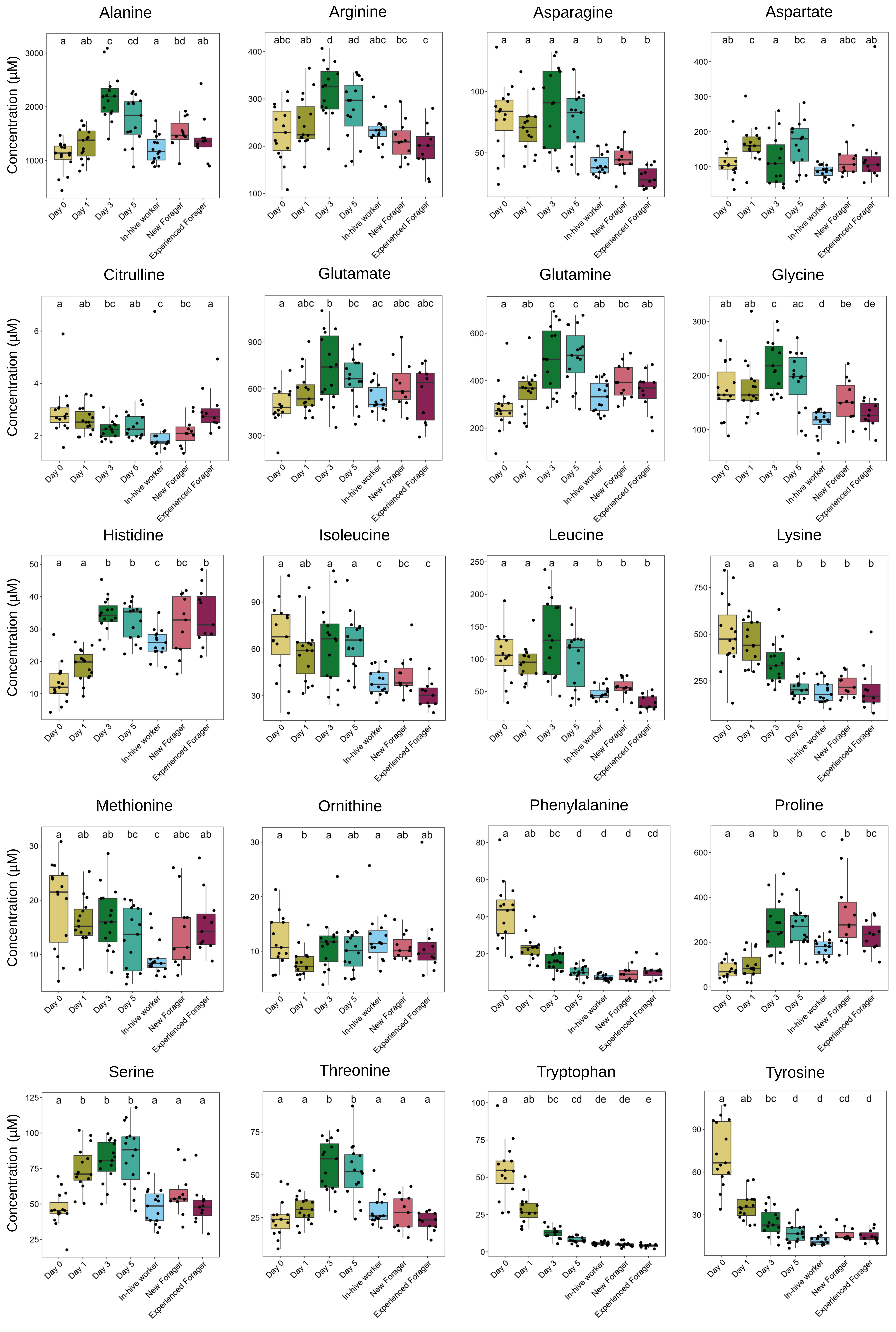

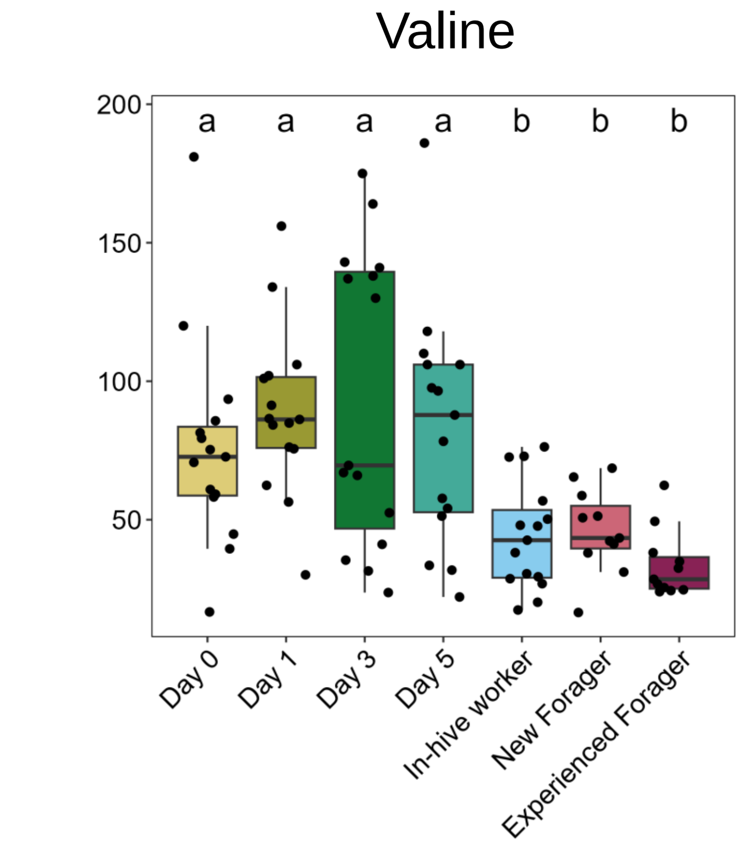

Supplementary Figure 5: Concentrations of all measured amino acids in the honeybee brains across all age groups. There are n = 15 samples for groups Day 0 to in-hive workers, and n =11 for new and experienced foragers. Significant changes between age groups were calculated using a Kruskal Wallis test followed by pairwise comparison using a Dunn's test. P-values were adjusted using the Benjamini-Hochberg method. Significance is shown using the compact letter display.

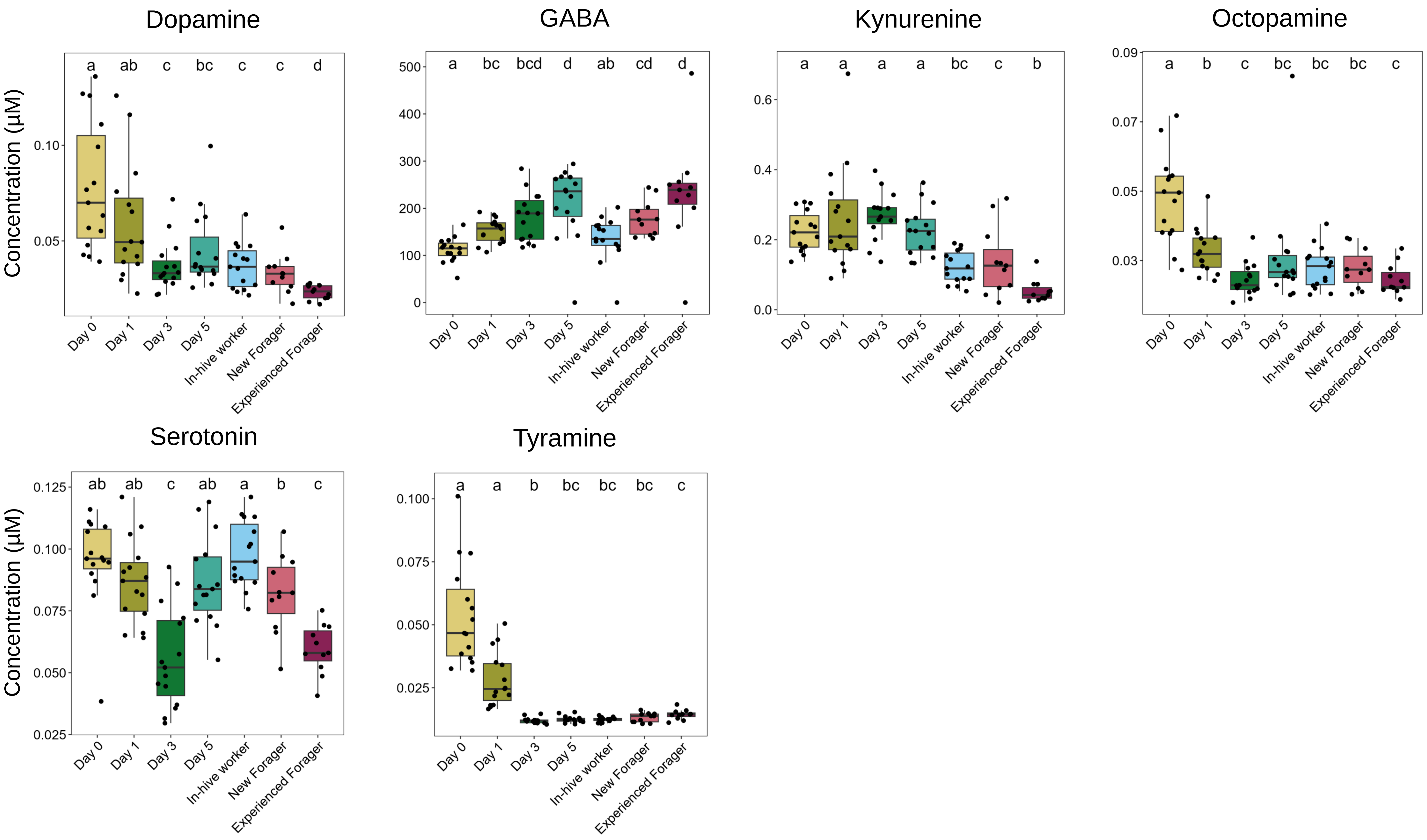

Supplementary Figure 6: Concentrations of all measured biogenic amines in the honeybee brains across all age groups. There are n = 15 samples for groups Day 0 to in-hive workers, and n = 11 for new and experienced foragers. Significant changes between age groups were calculated using a Kruskal Wallis test followed by pairwise comparison using a Dunn's test. *p*-values were adjusted using the Benjamini-Hochberg method. Significance is shown using the compact letter display.

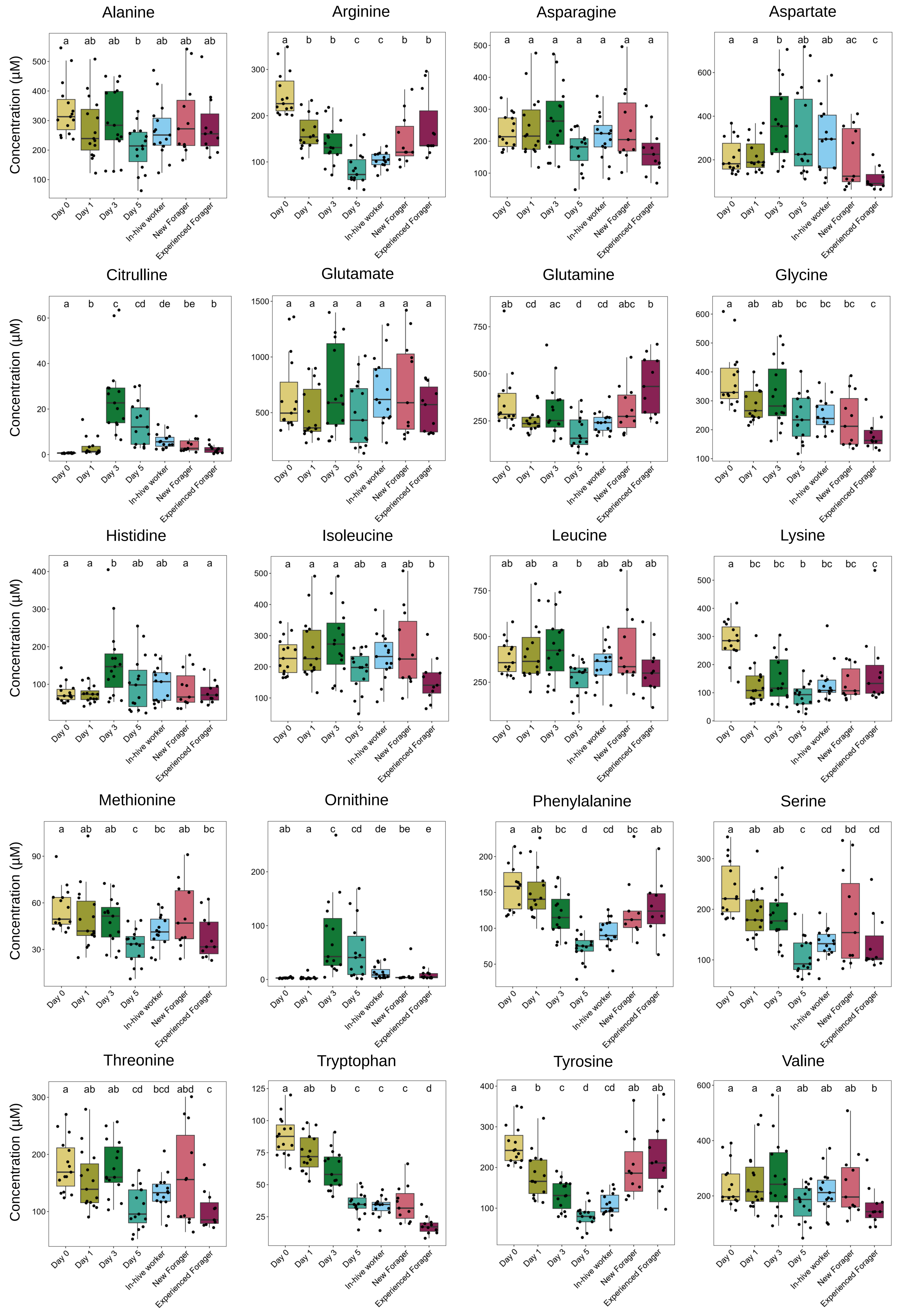

Supplementary Figure 7: Concentrations of all measured amino acids in the honeybee guts across all age groups. There are n = 15 samples for groups Day 0 to in-hive workers, and n = 11 for new and experienced foragers. Significant changes between age groups were calculated using a Kruskal Wallis test followed by pairwise comparison using a Dunn's test. *p*-values were adjusted using the Benjamini-Hochberg method. Significance is shown using the compact letter display.

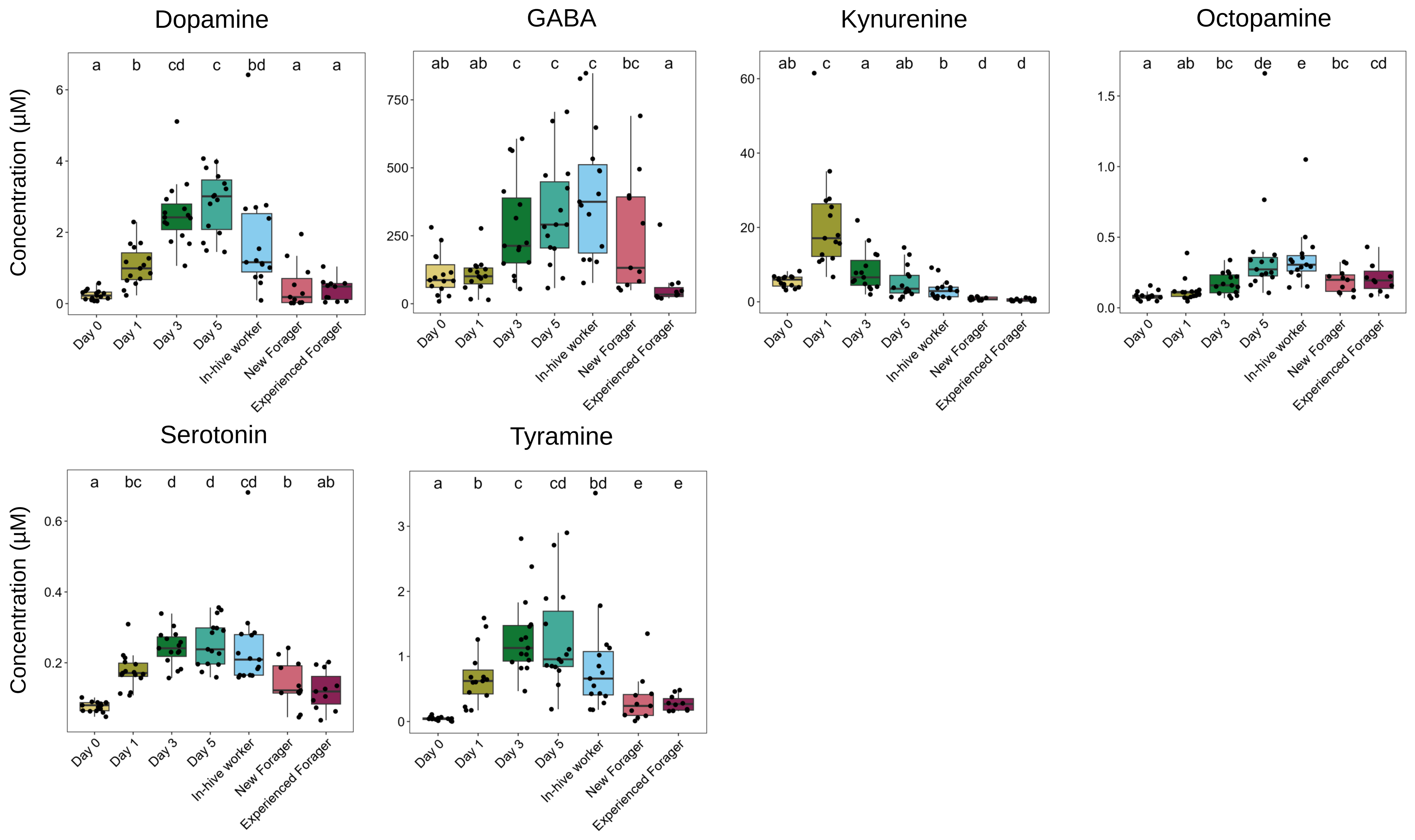

Supplementary Figure 8: Concentrations of all measured biogenic amines in the honeybee guts across all age groups. There are n = 15 samples for groups Day 0 to in-hive workers, and n = 11 for new and experienced foragers. Significant changes between age groups were calculated using a Kruskal Wallis test followed by pairwise comparison using a Dunn's test. *p*-values were adjusted using the Benjamini-Hochberg method. Significance is shown using the compact letter display.

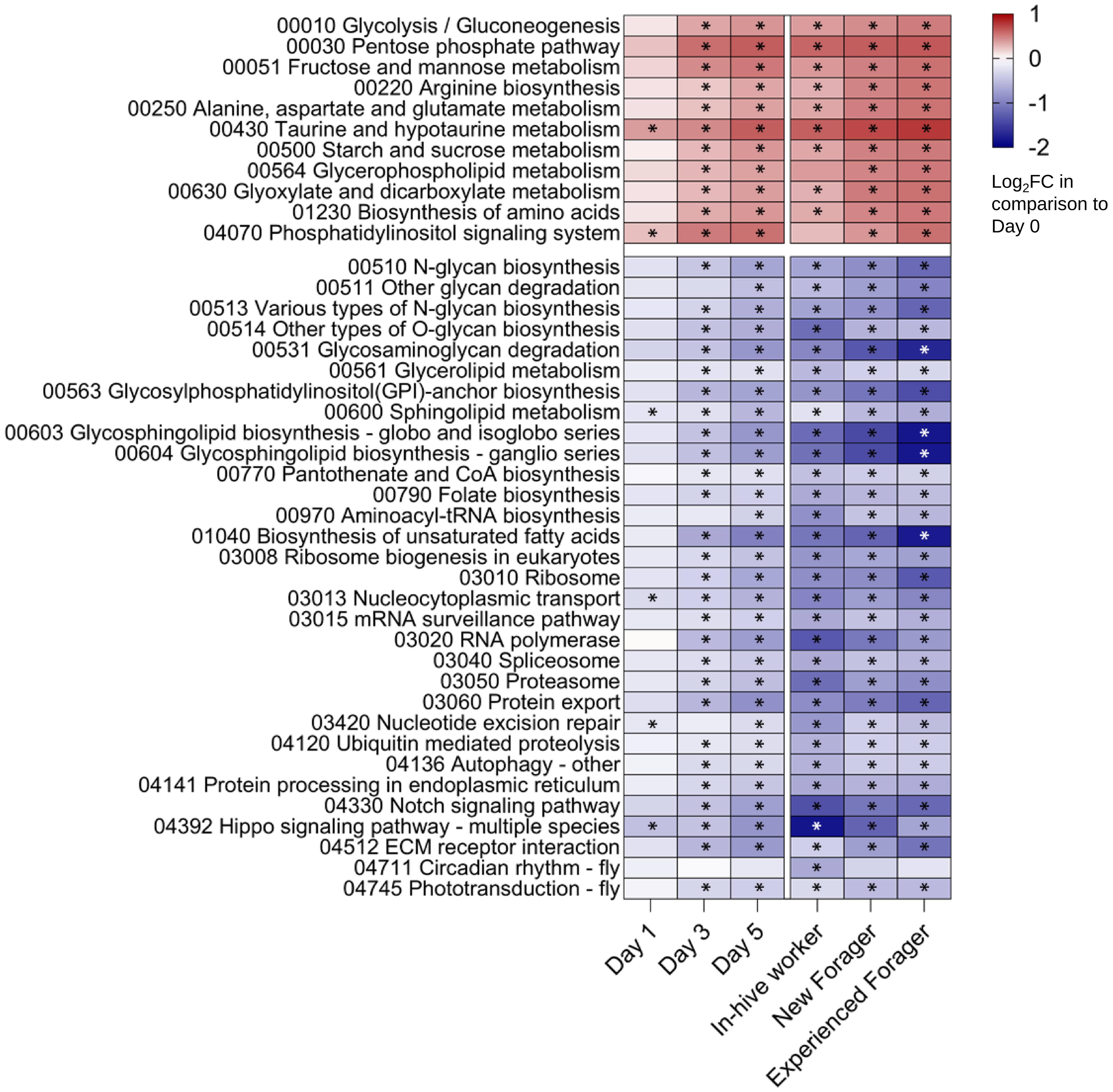

Supplementary Figure 9: Log_2 fold change (log_2FC) of a subset of detected KEGG pathways in the honeybee brains of all age groups in comparison to Day 0. All shown pathways show significant changes ($p < 0.05$) of at least one age group in comparison to Day 0 and a $log_2FC > |0.5|$. There are n = 15 samples for groups Day 0 to in-hive workers, and n = 11 for new and experienced foragers. Data underwent median-median normalisation prior to log_2FC calculations. Significant changes between age groups were calculated using a Kruskal Wallis test followed by pairwise comparison using a Dunn's test. *p*-values were adjusted using the Benjamini-Hochberg method. Significant differences to Day 0 are shown by the presence of asterisks *.

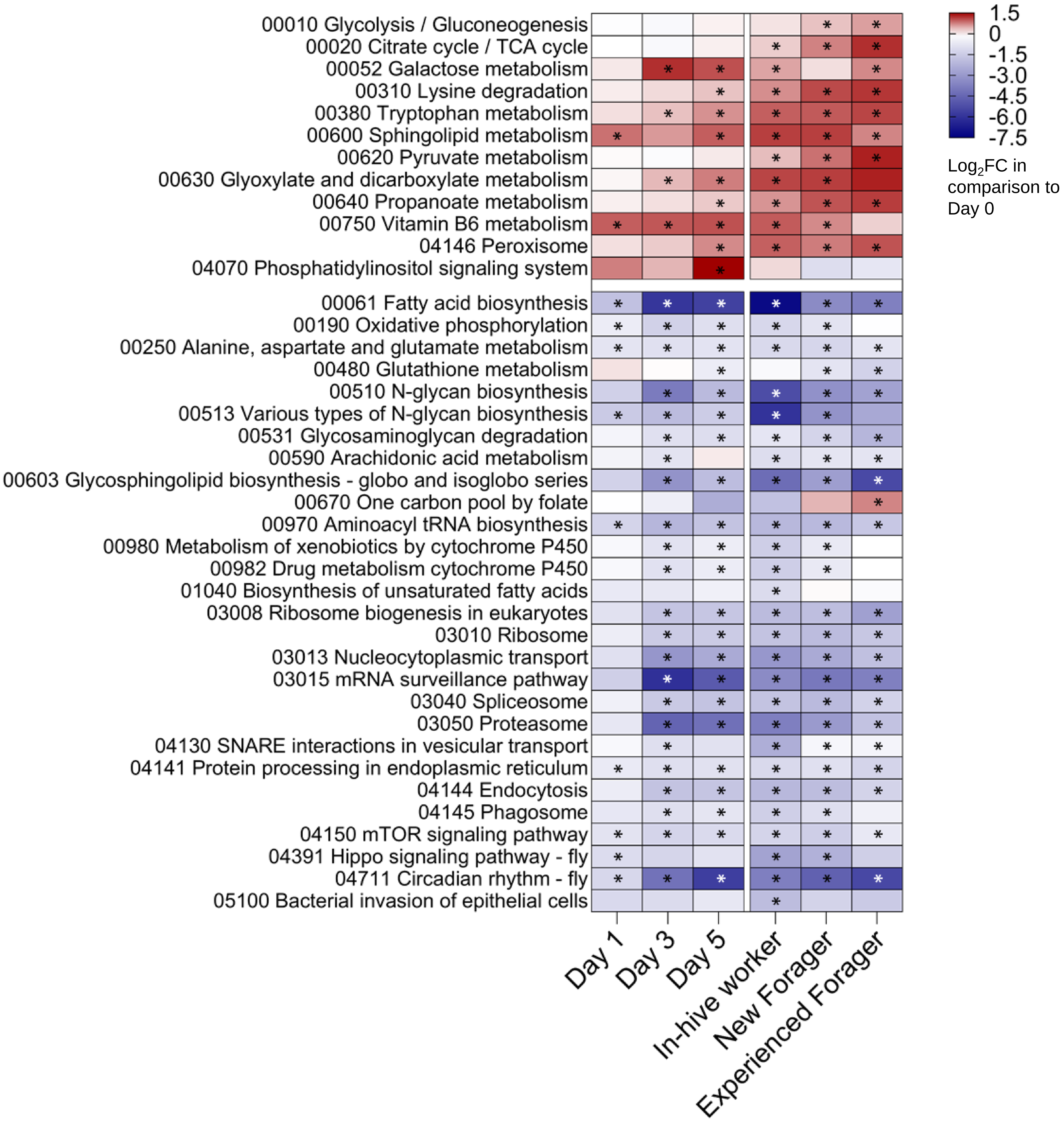

Supplementary Figure 10: Log_2 fold change ($\log_2$FC) of a subset of detected KEGG pathways in the honeybee guts of all age groups in comparison to Day 0. All shown pathways show significant changes ($p < 0.05$) of at least one age group in comparison to Day 0 and a $\log_2\text{FC} > |1|$. There are n = 15 samples for groups Day 0 to in-hive workers, and n = 11 for new and experienced foragers. Data underwent median-median normalisation prior to $\log_2$FC calculations. Significant changes between age groups were calculated using a Kruskal Wallis test followed by pairwise comparison using a Dunn's test. *p*-values were adjusted using the Benjamini-Hochberg method. Significant differences to Day 0 are shown by the presence of asterisks *.

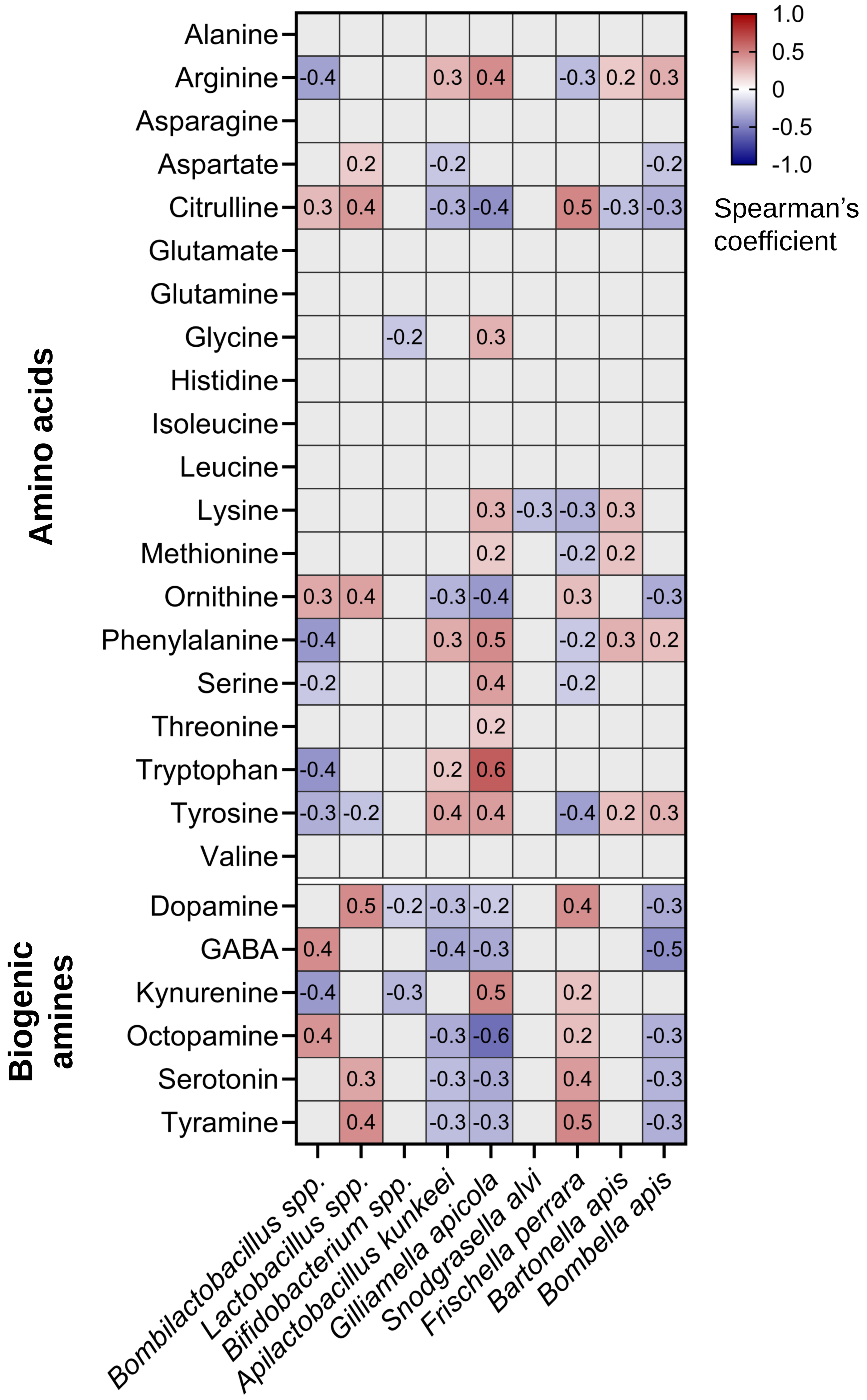

Supplementary Figure 11: Correlation analyses of bacterial phylotypes and amino acid and biogenic amine concentrations in the gut. Only significant correlations are shown. A Spearman's correlation was utilised and the relevant *p*-values were computed using algorithm AS 89 or via asymptotic *t* approximation in the *stats* package in *R*. The analysis is based on data from single samples (n = 97).

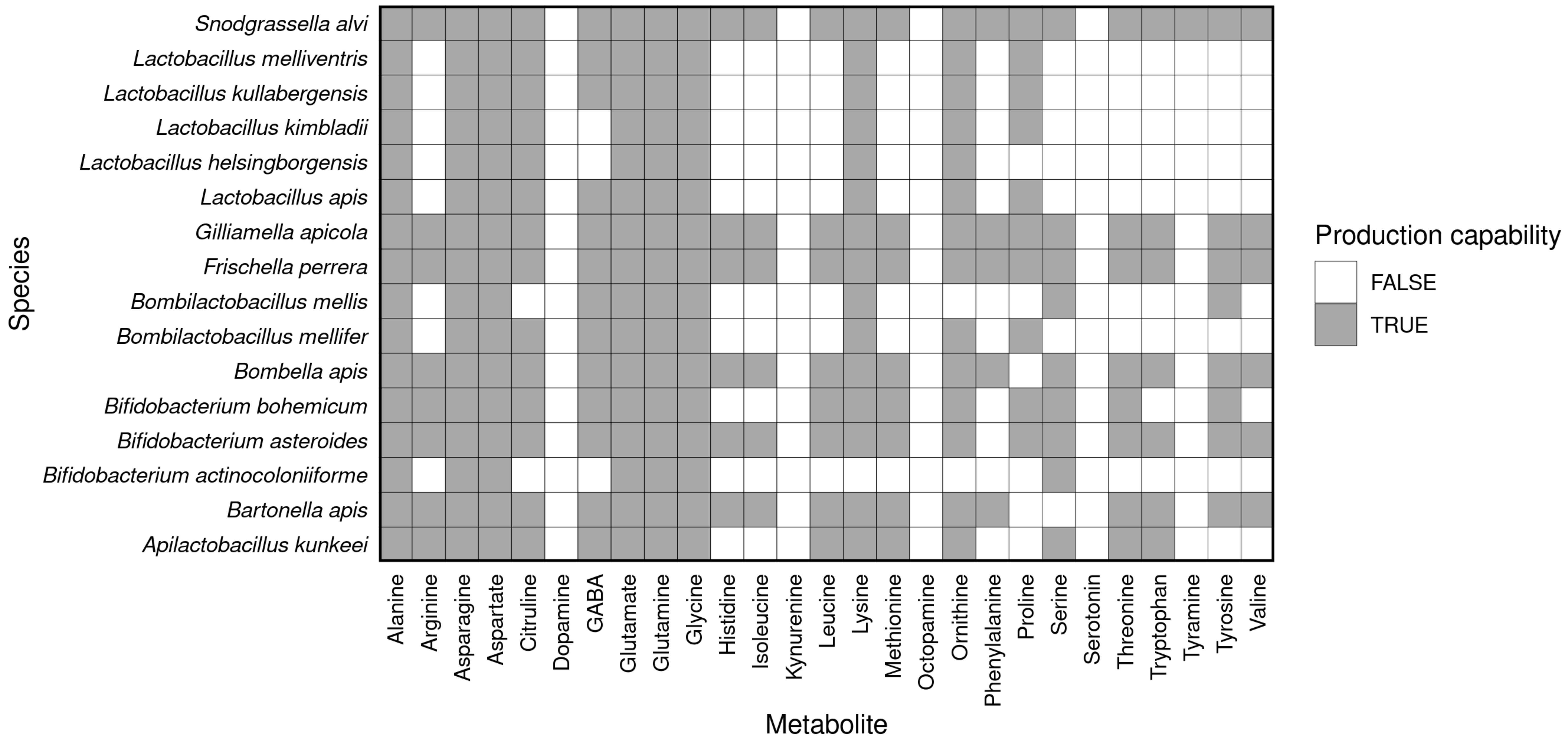

Supplementary Figure 12: Metabolite production capability of 16 honey bee gut bacterial species. Production capability was predicted using metabolic modelling based on genome-scale metabolic networks reconstructed by the software gapseq (https://github.com/jotech/gapseq).

Table S1: Isolation windows for MS/MS measurements in data independent acquisition mode. There are 48 individual scanning events, which comprise isolation windows of 24 *m/z* with a window overlap of 1 *m/z*.

Window	*m/z*	Window	*m/z*
1	349.4087-374.4200	25	925.6706-950.6820
2	373.4196-398.4309	26	949.6815-974.6929
3	397.4305-422.4419	27	973.6924-998.7038
4	421.4414-446.4528	28	997.7033-1022.7147
5	445.4523-470.4637	29	1021.7142-1046.7256
6	469.4632-494.4746	30	1045.7252-1070.7365
7	493.4741-518.4855	31	1069.7361-1094.7474
8	517.4851-542.4964	32	1093.7470-1118.7584
9	541.4960-566.5073	33	1117.7579-1142.7693
10	565.5069-590.5183	34	1141.76886-1166.7802
11	589.5178-614.5292	35	1165.7797-1190.7911
12	613.5287-638.5401	36	1189.7906-1214.8020
13	637.5396-662.5510	37	1213.8016-1238.8129
14	661.5505-686.5619	38	1237.8125-1262.8238
15	685.5615-710.5728	39	1261.8234-1286.8348
16	709.5724-734.5837	40	1285.8343-1310.8457
17	733.5833-758.5947	41	1309.8452-1334.8566
18	757.5942-782.6056	42	1333.8561-1358.8675
19	781.6051-806.6165	43	1357.8670-1382.8784
20	805.6160-830.6274	44	1381.8780-1406.8893
21	829.6269-854.6383	45	1405.8889-1430.9002
22	853.6379-878.6492	46	1429.8998-1454.9112
23	877.6488-902.6601	47	1453.9107-1478.9221
24	901.6597-926.6710	48	1477.9216-1502.9330

Table S2: List of species that were used to create the database against which the proteomic LC-MS/MS spectra from the honeybee guts and brains were searched. Brains were searched against the *Apis mellifera* database, whereas guts were searched against the *Apis mellifera* and Bacteria database. The bacterial species were chosen based on recent literature of honeybee gut microbiome sequencing grouped into their main phylotypes. Data from Uniprot release version 2024_02.

Phylotype	Included species	Organism ID (NCBI)	UniProt Number	Number of Entries
Apis mellifera				
Apis mellifera		7460	UP000005203	19,054
**Bacteria**				
Lactobacillus spp	*Lactobacillus apis*	303541	UP000033682	1,508
	Lactobacillus helsingborgensis	1218494	UP001164557	1,794
	Lactobacillus kullabergensis	1218493	UP000033533	1,865
	Lactobacillus kimbladii	1218506	UP000033612	1,890
	Lactobacillus melliventris	1218507	UP000033531	1,902
Bombilactobacillus spp	*Bombilactobacillus mellis*	1218508	UP000033695	1,572
	Bombilactobacillus mellifer	1218492	UP000033558	1,592
Bifidobacterium spp	*Bifidobacterium asteroides*	1437594	UP000224056	1,633
	Bifidobacterium bohemicum	1437606	UP000029096	1,631
	Bifidobacterium actinocoloniiforme	1437605	UP000029015	1,484
Apilactobacillus kunkeei		148814	UP000037778	1,332
Gilliamella apicola		1196095	UP000194977	2,770
Snograsella alvi		1196094	UP000019668	2,295
Frischella perrera		1267021	UP000030901	2,270
Bartonella apis		1686310	UP000187344	2,111
Bombella apis (previously *Parasaccharibacter apium)*		1510841	UP000027590	1,877

Table S3: Number of detected Master protein groups for each sample in each age group. Data is shown for bacterial proteins, honeybee gut and brain proteins.

Sample	Number of detected protein groups						
	Day 0	Day 1	Day 3	Day 5	In-hive worker	New Forager	Experienced Forager
**Bacteria**							
**1**	26	63	100	124	90	71	95
**2**	25	62	126	109	93	86	53
**3**	31	33	96	115	114	139	98
**4**	23	67	98	112	80	93	80
**5**	29	36	102	83	89	91	98
**6**	35	53	104	110	76	91	79
**7**	24	64	78	104	84	107	90
**8**	30	47	109	79	91	94	63
**9**	26	54	95	86	98	80	82
**10**	31	48	81	62	97	104	63
**11**	32	49	105	82	85	80	50
**12**	23	56	79	108	88		
**13**	33	53	97	113	127		
**14**	31	53	92	92	89		
**15**	30	34	48	111	83		
***Apis mellifera* gut**							
**1**	977	840	669	792	658	733	798
**2**	978	895	637	649	548	785	637
**3**	1082	757	605	664	441	657	632
**4**	1033	812	645	549	437	391	1013
**5**	849	789	611	639	511	489	423
**6**	1064	745	592	555	481	484	622
**7**	852	749	547	833	525	549	755
**8**	1067	728	562	596	509	619	563
**9**	960	793	627	687	557	511	598
**10**	953	666	497	499	545	409	453
**11**	853	724	555	509	485	646	505
**12**	882	678	504	468	547		
**13**	877	716	514	414	740		
**14**	902	719	502	579	585		
**15**	833	718	409	452	418		
***Apis mellifera* brain**							
**1**	3058	2987	3017	2899	2901	2942	2929
**2**	3070	3057	2999	2971	2923	2934	2945
**3**	3078	2994	3019	2975	2856	2933	2957
**4**	3037	3038	3035	2981	2912	2960	2890
**5**	3032	3007	2997	3011	2966	2959	2897
**6**	3015	3001	2968	2990	2888	2919	2947
**7**	3034	2997	2973	2963	2915	2922	2943
**8**	3036	3009	2962	2999	2754	2960	2941
**9**	3060	2865	3061	2911	2938	2917	2864
**10**	2981	2958	3000	2882	2876	2887	2954
**11**	2976	3046	2997	2930	2996	2980	2865
**12**	2977	2983	2976	2986	2778		
**13**	3035	2976	3012	2959	2975		
**14**	2969	3035	2968	2950	2945		
**15**	3069	3055	2891	2987	2968		
